# Genome-wide Association Study Reveals New Loci Associated With Pyrethroid Resistance in *Aedes aegypti*

**DOI:** 10.3389/fgene.2022.867231

**Published:** 2022-04-11

**Authors:** Luciano V. Cosme, José Bento Pereira Lima, Jeffrey R. Powell, Ademir Jesus Martins

**Affiliations:** ^1^ Department of Ecology and Evolutionary Biology, Yale University, New Haven, CT, United States; ^2^ Laboratório de Fisiologia e Controle de Artrópodes Vetores, Instituto Oswaldo Cruz/ FIOCRUZ, Rio de Janeiro, Brazil; ^3^ Instituto Nacional de Ciência e Tecnologia em Entomologia Molecular, INCT-EM, UFRJ, Rio de Janeiro, Brazil

**Keywords:** GWAS, kdr, vgsc gene, insecticide resistance, *Aedes*

## Abstract

Genome-wide association studies (GWAS) use genetic polymorphism across the genomes of individuals with distinct characteristics to identify genotype-phenotype associations. In mosquitoes, complex traits such as vector competence and insecticide resistance could benefit from GWAS. We used the *Aedes aegypti* 50k SNP chip to genotype populations with different levels of pyrethroid resistance from Northern Brazil. Pyrethroids are widely used worldwide to control mosquitoes and agricultural pests, and their intensive use led to the selection of resistance phenotypes in many insects including mosquitoes. For *Ae. aegypti*, resistance phenotypes are mainly associated with several mutations in the voltage-gated sodium channel, known as knockdown resistance (*kdr*). We phenotyped those populations with the WHO insecticide bioassay using deltamethrin impregnated papers, genotyped the *kdr* alleles using qPCR, and determined allele frequencies across the genome using the SNP chip. We identified single-nucleotide polymorphisms (SNPs) directly associated with resistance and one epistatic SNP pair. We also observed that the novel SNPs correlated with the known *kdr* genotypes, although on different chromosomes or not in close physical proximity to the voltage gated sodium channel gene. In addition, pairwise comparison of resistance and susceptible mosquitoes from each population revealed differentiated genomic regions not associated with pyrethroid resistance. These new bi-allelic markers can be used to genotype other populations along with *kdr* alleles to understand their worldwide distribution. The functional roles of the genes near the newly discovered SNPs require new studies to determine if they act synergistically with *kdr* alleles or reduce the fitness cost of maintaining resistant alleles.

## Introduction

Due to the lack of efficient vaccines for arbovirus such as dengue, Zika, and chikungunya, or limited vaccine supply for yellow fever, the use of chemical insecticides remains one of the main tools to control the mosquito-borne diseases (Dusfour et al., 2019). To control the anthropophilic mosquito *Aedes aegypti*, the primary vector of these viruses in many parts of the world (Souza-Neto et al., 2019), pyrethroid and organophosphate insecticides are widely used (McGregor and Connelly 2021) and have given rise to several populations with different levels of resistance across the world (Smith et al., 2016). In Latin America and especially in Brazil, pyrethroid resistance alleles are widespread (Melo Costa et al., 2020), and insecticide resistance is the main impediment to effective control of *Ae. aegypti*.

The leading cause of pyrethroid resistance are mutations in the voltage-gated sodium channel gene (*vgsc* or *Na*_*V*_), essential for neural stimulation (Zhorov and Dong 2017). These mutations cause changes in the gating kinetics of the sodium channels avoiding pyrethroid binding, which otherwise result in repetitive firing and/or membrane depolarization in the nervous system and death. Mosquitoes carrying a mutation in the *Na*_*V*_ gene maybe be entirely resistant to pyrethroids or suffer knockdown but can recover. The knockdown resistance (*kdr*) was first reported in house flies (Busvine 1951) and is now found in several arthropods due to the intensive use of pyrethroids insecticides. However, even within *Ae. aegypti,* there are significant differences in the resistance levels among populations and which mutations they have. The rise and spread of *kdr* mutations likely occurred independently throughout the world (Cosme et al., 2020; Fan et al., 2020), and their persistence over time varies (Macoris et al., 2018; Vera-Maloof et al., 2020) due to their fitness cost in the absence of pyrethroid use (Rigby et al., 2020; Smith et al., 2021).

Recent studies using single populations revealed the enrichment of several genes (Campbell et al., 2019) or single nucleotide polymorphisms (Saavedra-Rodriguez et al., 2021) in pyrethroid-resistant mosquitoes, indicating the involvement of other physiological selected mechanisms, which could be involved in increased detoxification of insecticides and cuticle impermeability, inhibiting insecticide penetrance. However, mosquitoes collected from the field may be exposed to different environmental chemicals that can potentially affect the expression levels of genes involved in detoxification processes. Local adaptation and drift may also affect the results of genome-wide association studies (GWAS) using only one homogenous population from the same genetic group, even though their pyrethroid resistance is heterogeneous. Recent analytic tools can detect and adjust for confounding complexity associated with a specific trait and natural genetic variation. For example, principal component analysis (PCA), multidimensional scaling (MDS), or genetic relatedness matrix (GRM) (Yang et al., 2011; Chang et al., 2015; Hellwege et al., 2017).

To help us to understand the pyrethroid resistance mechanisms in *Ae. aegypti* additional to *kdr*, our study aimed to use classical GWAS with two *Ae. aegypti* populations from different cities in Northern Brazil while controlling for population stratification. These two populations were heterogeneous in pyrethroid resistance and had different *kdr* allele frequencies, and haplotypes (Cosme et al., 2020). Oiapoque mosquitoes have a *kdr* haplotype also found in Asia, while Macapa has a haplotype found only in South America. They were also phenotypically different, with varying levels of susceptibility, knockdown, and complete resistance. We used the *Ae. aegypti* SNP-chip (Evans et al., 2015) to genotype the G0 insects from the field and qPCR to check their *kdr* alleles.

We hypothesized that SNPs in genes closely linked to *Na*_*V*_ could also be associated with pyrethroid resistance due to a selective sweep, or SNPs in genes far from the *vgsc* could act synergistically (epistatically) with the *kdr* alleles to increase metabolization of insecticides or decrease the fitness cost of having such alleles. Besides, introgression of genomic regions from chromosome 3 with the resistance *kdr* alleles can result in changes in the genomic architecture which can be observed by changes in linkage disequilibrium. Finally, two-locus interaction analysis could identify epistatic pairs that would also act synergistically with other loci directly related to pyrethroid resistance. Since we used two populations with only slightly different genetic backgrounds, we did not expect to find such loci.

## Materials and Methods

### Mosquito Collections

**FIGURE 1:**
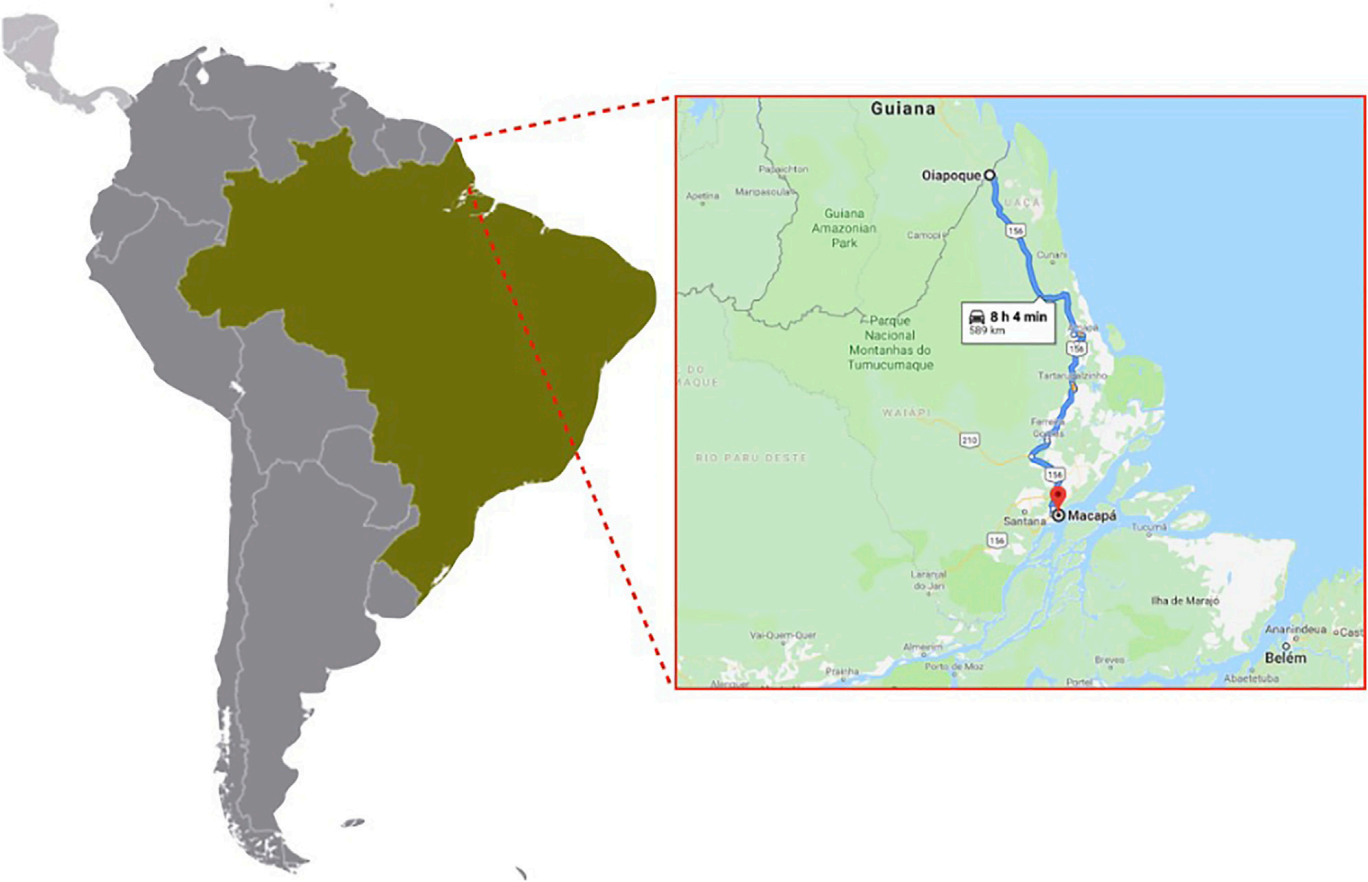
Location of our egg collection sites, Oiapoque and Macapa, Amapa, Brazil. Map, *Google Maps.* Accessed 9 June 2021.

We collected mosquitoes from two cities of the northern Brazilian state Amapa in January 2015 (Figure 1). Briefly, we collected mosquitoes’ eggs during three consecutive weeks in Macapa and Oiapoque cities using 60 ovitraps in a grid of 500 × 500 m^2^ per locality. We brought the ovitraps’ paddles to the laboratory, hatched the eggs, and reared the mosquitoes in standard conditions (12 h:12 h light-dark, 70% relative humidity, 27°C). We refer to mosquitoes collected in each city as “populations” in this study.

### Insecticide Exposure Bioassay

**FIGURE 2:**
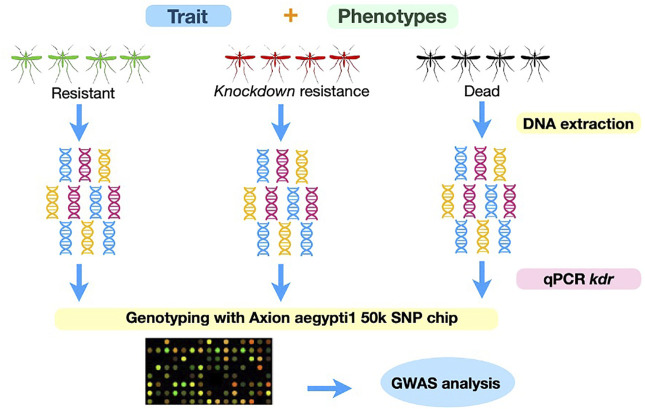
Experimental design. We exposed mosquitoes from each population to deltamethrin, via impregnated papers in tubes, for one hour. We kept females that were alive in −80^o^C for DNA extraction. We stored the mosquitoes that were not moving for 24 h to separated knockdown resistant mosquitoes from susceptible (dead). After 24 h, we divided the mosquitoes that survived the exposure but were knocked down from the dead. We conducted three biological replicates. We extracted DNA from all mosquitoes simultaneously, genotyped them for the *kdr* resistance alleles by qPCR, and preceded with genotyping using the Axion *aegytpti1* SNP chip.

We used an adaptation of the World Health Organization (WHO) test tubes bioassays described elsewhere (Brito et al., 2018). Mosquitoes from Oiapoque are considerably more resistant to pyrethroids than mosquitoes from Macapa (Salgueiro et al., 2019) Therefore, we used different doses for each population to obtain the following phenotypes: resistant, susceptible, and knockdown resistance (Figure 2). We exposed mosquitoes from Oiapoque and Macapa to papers impregnated with 1.2 g/cm^2^ and 0.6 g/cm^2^ of deltamethrin, respectively, following the methodology described previously (Brito et al., 2018). Next, we removed the females alive (resistant) and kept them at −20°C until DNA extraction. We transferred the remaining females to new clean tubes for 24 h. The goal was to separate the females with knockdown resistance and susceptibility. Finally, we separated the females alive and dead and kept them at -20°C until DNA extraction.

### DNA Extraction

We extracted total nucleic acids from all individuals using the DNeasy Blood and Tissue kit (Qiagen), following the manufacturer’s instructions, and eluted the samples in 200 µl of 1% TE buffer. We performed an additional step treating our samples with 4 µl of RNase A (Qiagen). We stored all samples at −20°C until further analysis.

### *Kdr* Genotyping

We performed independent genotyping reactions for each *kdr* site based on a qPCR approach using the Custom TaqMan SNP Genotyping Assay (ThermoFisher) (Macoris et al., 2018) (see Supplementary Table S1 for the primer and probe sequences for each assay). Reactions consisted of 1X TaqMan Genotyping Master Mix (ThermoFisher), 1X of the respective Custom TaqMan SNP Genotyping Assay, 20 ng of DNA and ultra-pure water q. s. 10 µl, run in a QuantStudio 6 Flex (Applied Biosystems), under standard conditions: 45 cycles with a DNA denaturation step (95°C for 15 s) and primer and probe annealing, followed by DNA polymerization (60°C for 1 min). The genotypes were obtained by the online software Genotype Analysis Module V3.9 (Applied Biosystems, Thermo Fischer cloud platform). We evaluated the *kdr* sites 1016 (V1016I) and 1534 (F1534C) in both populations and genotypes were determined as detailed elsewhere (Macoris et al., 2018) (Supplementary Table S2).

### SNP Genotyping

We used the custom-designed SNP chip for *Ae. aegypti* to genotype all mosquitoes (Evans et al., 2015). Once we selected the mosquitoes with the desired phenotype, we removed the DNA samples from the −20°C freezer to concentrate and purify them using Amicon^R^ Ultra 30k centrifugal filter devices (Millipore) according to the manufacturer instructions. We obtained approximately 23 µl of eluting. Next, we checked the genomic DNA concentration using Qubit (Invitrogen). Finally, we normalized the DNA concentrations to 20 ng/μl and sent approximately 200 ng of genomic DNA from individual mosquitoes to the Functional Genomics Core at the University of North Carolina, Chapel Hill, for hybridization with the Axiom aegypti1 SNP chip (Life Technologies Corporation CAT#550481). We used the Affymetrix Genotyping Console v.3.1.51.0 (Affymetrix) to generate and process the genotype calls (Evans et al., 2015). Briefly, we used the default parameters outlined as best practice for non-human samples, except for the call threshold set to 90%, and by using the off-target variant correction. We genotyped a total of 95 individuals using the AaegL5 genome assembly. The SNP chip was developed in the previous assembly with approximately 3.7 thousand scaffolds. Once the chromosomal level physical mapping was available, the probes were mapped to the AaegL5 and the annotation was lifted, resulting with approximately 46 thousand SNPs on the AaegL5 genome assembly. Probes mapping multiple sites were not included in the new Affymetrix libraries.

### Quality Control

We conducted stringent quality control (QC) steps before the association analysis outlined elsewhere (Marees et al., 2018) using Plink v. 1.90b6.2 (Chang et al., 2015). Briefly: 1) we removed SNPs missing in more than 10% of individuals (missing in nine mosquitoes); 2) we removed individuals with missing genotypes calls higher than 10% of the SNPs; 3) we used a minor allele frequency threshold of 10%; 4) we excluded markers which deviated from Hardy-Weinberg equilibrium (HWE), filtering HWE *p-value* < 1e^−6^ for continuous phenotype; 5) we excluded individuals with high or low heterozygosity rates (±3 SD from mean), which could indicate sample cross-contamination; 6) we calculated the identity-by-descent (IBD) of all mosquitoes pairs used only founders; 7) we used independent autosomal SNPs (pruning using--indep-pairwise 50 kb 1 0.2) for principal component analysis (PCA) and for the multidimensional scaling (MDS) approach to evaluate clustering and remove outliers. The MDS method calculates the genome-wide average proportion of alleles shared between any pair of individuals within the sample to generate quantitative indices of each mosquito’s genetic variation. We explored the MDS and PCA plots to verify clustering in our samples and removed any mosquito outlier. We evaluated the relationships among the individuals *via* the Plink Z-values. A Z0 value of approximately one indicates completely unrelated individuals, while a Z2 value near one indicates identical samples or twins.

### Genotype Imputation

In our quality control we removed SNPs missing in more than 10% of the individuals. Imputing the genotypes of this missing SNPs will increase the power of our GWAS. Differently from human and other species where a golden standard SNP reference panel is known, such panel is not available for *Ae. aegypti*. Another barrier is the SNP density required for genotype imputation, which is at least 200 SNPs/Mb in humans (Shi et al., 2017). We do not know the minimal required density for *Ae. aegypti* but it is probably lower since the linkage patterns are different, with *Ae. aegypti* displaying large linkage blocks across the genome (Evans et al., 2015). Finally, due the nature of the SNP chip dataset, we are not able to phase the SNPs and perform the genotype imputation based on the haplotypes. Because the chip was designed not considering the strand of each SNPs, and since its release the annotation was lifted to the current genome assembly, it is extremely difficult to phase haplotypes from the SNP chip data. Finally, our sample size is small to accurate determine all possible haplotypes to then impute the missing genotypes based on these haplotypes.

### Association Analysis

Our GWAS aimed to identify SNPs with allele frequencies varying systematically as a function of the pyrethroid resistance. We categorized our phenotypes into three groups: susceptible, resistant, and knockdown resistance. We considered mosquitoes that did not suffer the knockdown effect of deltamethrin, i.e., were alive after 1 h of exposure and remained so 24 h after, as the resistant phenotype. We counted mosquitoes that were alive and did suffer the knockdown effect 24 h after the exposure to deltamethrin as the resistant phenotype. Finally, we regard mosquitoes that died 1 h of exposure to deltamethrin and did not recover 24 h after as the susceptible phenotype.

To consider the population stratification we observed in our dataset, we used dimensional reduction using multidimensional scaling (10 components) as covariates to account the population structure (Plink option “--covar”).

We perform association tests with PLINK (Chang et al., 2015), treating the insecticide resistance trait as quantitative. We used the option--linear to perform linear regression analysis using an additive model with each SNP as a predictor. Next, we performed the Benjamini-Hochberg false discovery rate (FDR) correction for multiple testing using PLINK. Since FDR does not imply statistical significance and is used only to decrease false positives, we applied Bonferroni correction. Bonferroni is more conservative and controls the probability of having at least one false-positive finding. It allows us to control the expected proportion of false-positive among all signals using an FDR threshold of 0.05, assuming all SNPs are independent (Benjamini and Hochberg 1995).

We also used the software Genome-wide Complex Trait Analysis (CGTA) (Yang et al., 2011) to perform the association analysis, by estimating the variance explained by all the loci on the genome for a specific trait instead of testing the association of a particular locus to the trait in question. CGTA uses a mixed linear model analysis of variance explained by the SNPs. We performed a mixed linear-based association analysis with our LD pruned data and used the same covariates from the Plink analysis.

We performed sliding-window Fst (Weir and Cockerham 1984) estimates between resistant plus knockdown versus susceptible mosquitoes from both populations. We calculated the Weir and Cockerham weighted Fst values for 1 Mb windows in the genome with 100 kb steps using vcftools (Danecek et al., 2011).

We sought to find other loci linked with the those associated with the pyrethroid resistance by performing pairwise linkage disequilibrium (LD) measurements of *D’* and *r* ^2^ (Lewontin 1964; Hill and Robertson 1968) using the package LDBlockShow (Dong et al., 2020). Next, we performed epistasis (Wilson 1902; Fisher 1919) analysis to look for other SNPs with distortions from Mendelian segregation ratios because of the SNPs we identified as associated with pyrethroid resistance. We assumed that the loci we found in our GWAS must have a biological interaction. We considered epistasis as a departure from a linear model in which the phenotypic effects of genotypes at two or more loci are assumed to be additive. We performed the analysis using Plink (Chang et al., 2015). We estimated the frequency of the genotypes of the newly discovered SNPs affected by the significant SNPs from our study.

To explore the effects of the introgression of the *kdr* alleles or the genomic regions linked to the *kdr* mutations into the chromosome 3 we looked at LD patterns of resistant and susceptible mosquitoes. We subset the data from each population (OAIs = Oiapoque susceptible, OIAr = Oiapoque resistant, MACs = Macapa susceptible, and MACr = Macapa resistant), and calculated the pairwise *r* ^2^ values (Hill and Robertson 1968) for all SNPs with minor allele frequency equals to 5% within each group. We focused on chromosome 3 where the *kdr* mutations are located. We generate the LD matrices with Plink (Chang et al., 2015) and used LDna (Kemppainen et al., 2015) to find single outliers clusters of SNPs. We tested several values of phi (*φ*) and the number of edges (|*E*|_min_) to form each LD cluster for all populations (*φ* = 2 to 8, and the |*E*|_min_ = 10–60), and used SNPs present in all groups with missing genotypes. We chose a set parameter and used it for all groups, since using different parameters for each group could generate different LD clustering patters. Finally, we estimated the size of each LD cluster. Due to interspersed or mosaic-like LD patterns found, we only plotted clusters bigger than 1 Mb in size using the R package Sushi v. 1.28.0 (Phanstiel et al., 2014).

### GWAS Power Analysis

The goal of our study was to discover other genomic regions acting synergistically with the known *kdr* mutations. As our phenotypic dataset shows, some individuals carrying susceptible alleles do not die or get knockdown after exposure to pyrethroids, indicating unexplained genetic variance. The statistical power of our statistical significance test is the probability that the test will reject the null hypothesis H0 at the given significance threshold when the data follow a specific alternative hypothesis H1. In our GWAS, the H1 is specified by fixing the sample size (N = 90 after quality control) and parameters describing the variants, as minor allele frequency (MAF) and effect size. Since not all true effects are the same, we represent our power analysis as a power curve over a range of parameters values.

## Results

### Insecticide Exposure Bioassay

**FIGURE 3:**
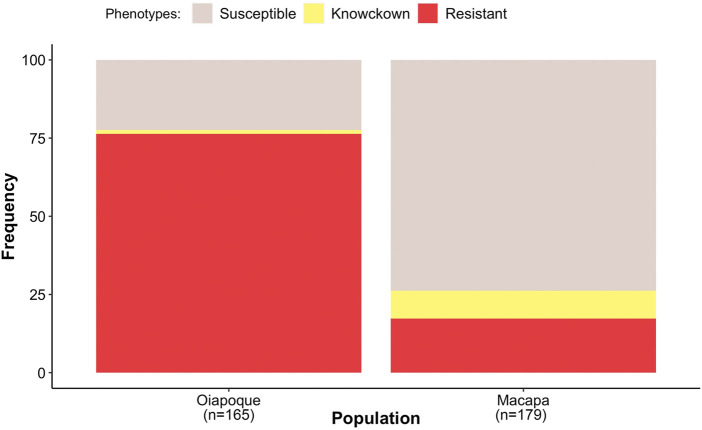
The proportion of the phenotypes within each population. Resistant (mosquitoes alive after 1-hour of the exposure), knockdown resistance (mosquitoes were knocked down 1 h after exposure but were active 24 h later), and susceptible (mosquitoes died after 1 h of exposure and were not active 24 h later). We genotyped 95 individuals for our GWAS. See Supplementary Table S3 for more details.

**FIGURE 4:**
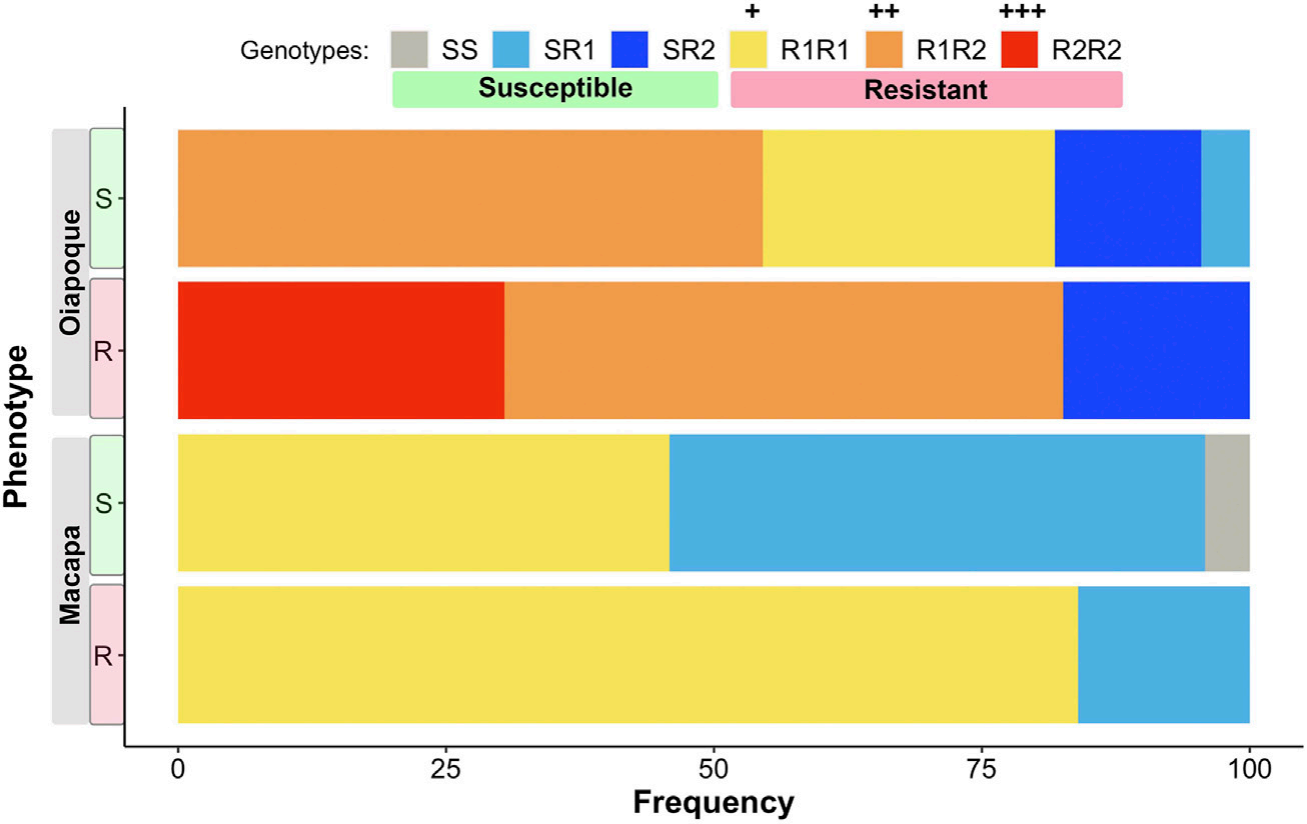
The proportion of genotypes for each population. Two phenotypes: Resistant (R)—mosquitoes that did not die 24 h after exposure, and Susceptible (S)—mosquitoes were dead 24 h after the exposure. Mosquitoes with the genotypes SS, SR1, and SR2 are phenotypically susceptible. Mosquitoes with the genotypes R1R1, R1R2, and R2R2 are phenotypically resistant, but the resistance levels are different, with the R2R2 genotype giving the highest level of resistance. Mosquitoes were genotyped via qPCR using primer and probes described in Supplementary Table S1.

**FIGURE 5:**
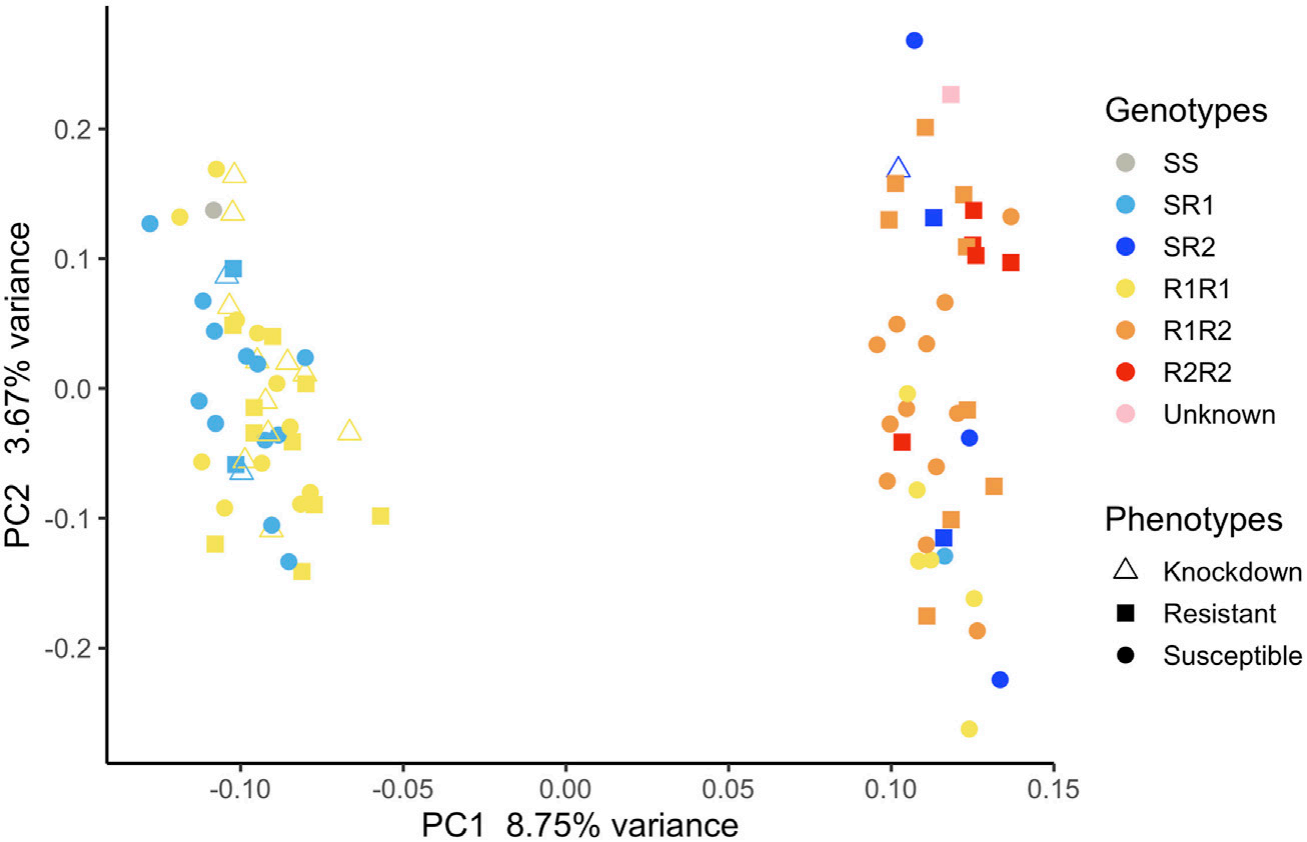
Principal component analysis with all the mosquitoes used in our association analyses. Each mosquito was genotyped using qPCR to identify each *kdr* genotype they had.

Mosquitoes from Oiapoque and Macapa displayed different levels of knockdown and complete resistance. We exposed mosquitoes from Oiapoque to deltamethrin with a concentration two times higher than Macapa, 1.2 and 0.6 mg/L respectively. However, most Oiapoque mosquitoes (76%) were alive 1 h after the exposure, contrasting with Macapa mosquitoes, where less than a quarter were active (Supplementary Table S3). Only two Oiapoque mosquitoes displayed knockdown resistance, recovering after knockdown (1%), whereas 32 Macapa mosquitoes displayed knockdown resistance (9%). At 24 h after the initial exposure, most of the Macapa’s mosquitoes died (74%), but most of the Oiapoque mosquitoes survived (22% mortality) (Supplementary Table S3; Figure 3). The *kdr* R1 allele (V1016 + 1534C) was present in almost all mosquitoes from Macapa (95.83%) and the genotype R1R1 was more frequent in the resistant (45.83%) than in susceptible (4.17%). The *kdr* R2 allele (1016I + 1534C) was absent in Macapa, but found in all resistant mosquitoes from Oiapoque, with the homozygous genotype R2R2 found only in the resistant mosquitoes, while in Macapá and the genotype R1R1 was more frequent among the resistant mosquitoes. Most of the mosquitoes from Macapa had *kdr* genotypes (R1R1) associated with pyrethroid resistance. In contrast, mosquitoes from Macapa had a higher frequency of the genotypes related to high levels of resistance (R1R1, R1R2, and R2R2), justifying the higher resistance of Oiapoque (Figure 4 and Supplementary Table S9). Indeed, all Oiapoque mosquitoes survived the exposure to 0.6 mg/L (data not shown). Our PCA analysis also indicated no clustering by *kdr* genotype within each population (Figure 5). Only one mosquito genotyped with the chip did not have any *kdr* allele, and all the others were carrying at least one allele. In the SNP-chip, there are probes for 11 loci on the *vgsc* gene. However, most of them were filtered out due to minor allele frequency, indicating near fixation of *kdr* alleles in these populations.

### Association Analysis

We obtained 40,800 variants genotyped across our 95 samples. We removed 6,819 markers that were not present in at least 90% of the individuals (Plink flag “--geno 0.1”), remaining with 33,981 variants (Supplementary Figure S1A). We removed three individuals that did not have at least 90% of the markers (Plink flag “--mind 0.1”) (Supplementary Figure S1B) and removed 1,222 markers from genomic regions not assigned to the autosomal chromosomes. Next, we removed 18,251 variants with minor allele frequency (MAF) smaller than 10%, obtaining 14,508 variants (Supplementary Figure S1C), and removed variants not in Hardy-Weinberg equilibrium (HWE) for each population separately, drawing a total of 352 variants (plink flag “ --hwe 0.000001”, Supplementary Figure S2). Next, we performed linkage disequilibrium-based variant pruning before heterozygosity estimation (Plink flag “--indep-pairwise 50 kb 1 0.2”). We removed three individuals whose heterozygosity rate deviated more than three standard deviations from the heterozygosity rate mean (Supplementary Figure S3D).

High levels of relatedness among individuals could potentially influence PCA, other population-based estimates, and our association studies. Accordingly, we assessed the background relatedness in our dataset to ensure the robustness of various genetic inferences. We performed identity-by-descent (IBD) estimates between pairs of individuals using the LD-pruned data. The Plink average PI_HAT estimate was 0.02, indicating low levels of cryptic relatedness in our dataset (Supplementary Figure S3A,B). After QC, we obtained 90 samples used in our association analysis.

Among all possible pairwise comparisons among the mosquitoes in our dataset, 92.3% resulted in Z0 above 0.9, meaning they are unrelated. Most of the Z2 estimates were below 0.01 (90.9%), indicating that our samples were unrelated (Supplementary Figure S3C). We did not find any evidence of duplicates, parent-offspring, or monozygotic twins in our dataset.

**FIGURE 6:**
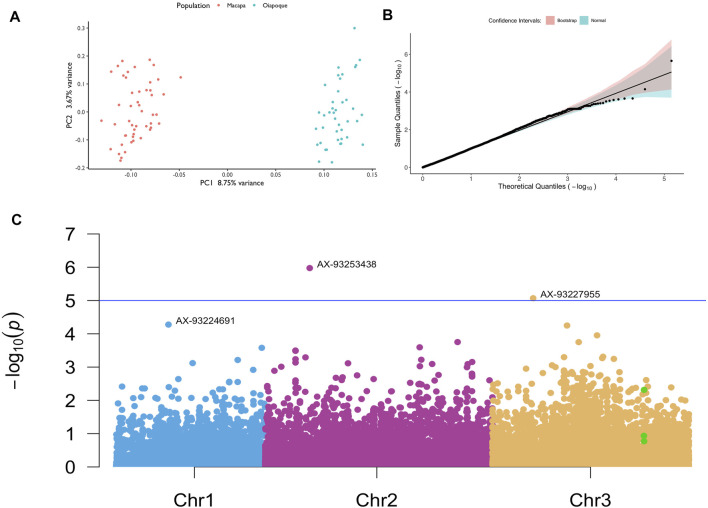
GWAS in mosquitoes with different levels of knockdown resistance. **(A)** Principal component analysis showing two different clusters; **(B)** Quantile-quantile plot with confidence intervals; **(C)** Manhattan plot with the SNPs with the lowest *p* values annotated per chromosome. Green dots are SNPs on the *vgsc* gene. The SNP chip has 11 SNPs on the *vgsc* gene; only 3 SNPs passed our filtering, with most being removed due to low minor allele frequency.

Using the autosomal LD-pruned variants to check for the presence of subpopulations in our study, the principal component analysis showed two clusters in our dataset, one for each population (Figure 6A).

Our power analysis revealed that although our sample size is small after QC (N = 90), we still have power to detect significant variants depending on the effect size of each variant (Supplementary Material S1). Although we do not know the variants true effect, we tested values varying from 0.2 to 0.6. It is worth to remember that a single *kdr* mutation results in a complete resistant phenotype with a large effect size. We were able to find two variants above the significance threshold of 5e-5 and after correction of multiple testing. These loci and others that we did not have the power to detect may be acting synergistically with *kdr* mutations that do not confer completely resistance to pyrethroids, helping to detoxify the neurons from the insecticide. A higher sample size and more variants is necessary to completely rule out the contribution of other genes towards resistance.

We observed a uniform distribution of the uncorrected *p* values, indicating that a small percentage of our hypothesis was non-null (Supplementary Figure S1D). After corrections for multiple testing, we found two SNPs above our significance threshold, associated with the KDR resistance in *Ae. aegypti* (Supplementary Table S4; Figure 6B,C). Our association analysis using linear mixed models with CGTA lead to the same SNPs with the lowest unadjusted *p* values (Supplementary Table S5).

**FIGURE 7:**
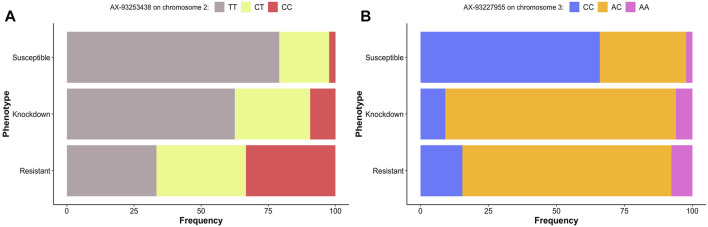
Genotype’s frequency (%) of the alleles associated with pyrethroid resistance in *Ae. aegytpi.* Most homozygous mosquitoes carrying the allele T, loci AX-93253438 **(A)**, and C, loci AX-93227955 **(B)**, died within one hour of exposure to deltamethrin.

Approximately 79% of the homozygous mosquitoes for the allele T for loci AX-93253438 in chromosome 2 were susceptible to deltamethrin (Figure 7A). The frequency of heterozygotes and homozygotes for allele C for the same loci increased in resistant and knockdown mosquitoes. However, about 33% of homozygous for allele T were resistant to deltamethrin (Figure 7A).

The loci AX-93227955 in chromosome 3 genotype frequencies indicated that about 66% of homozygous mosquitoes for allele C were susceptible to deltamethrin (Figure 7B). For the same locus, the proportion of heterozygotes and homozygotes for allele A is similar in resistant and knockdown mosquitoes, with most mosquitoes being heterozygous.

**FIGURE 8:**
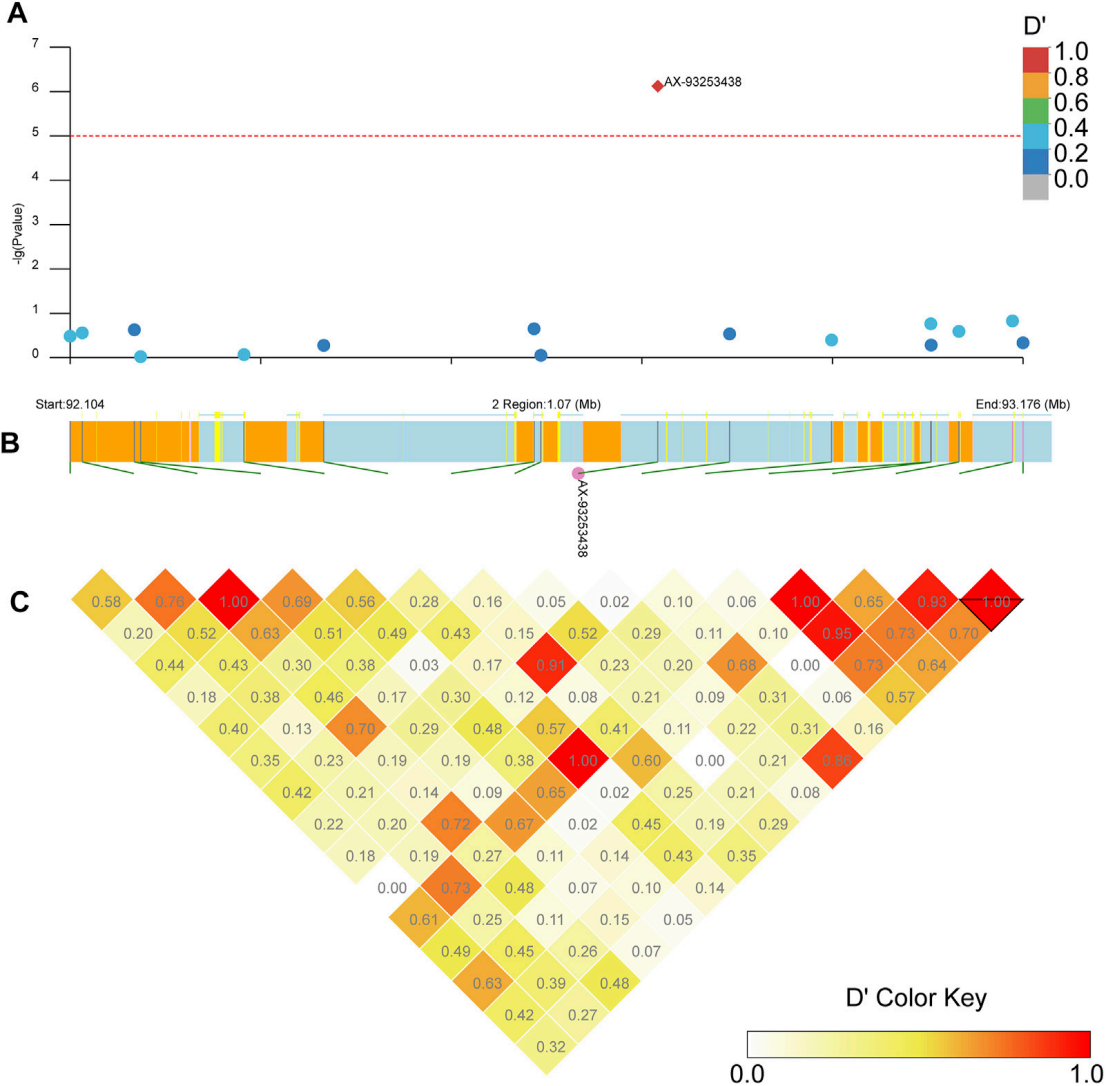
Linkage block analysis of loci AX-93253438 on *Ae. aegypti* chromosome 2 using the R package LDBlockShow. **(A)** Manhattan plot with significance line (red). SNPs are colored following the key at top right. **(B)** Genes in the region with SNPs marked as green lines, where the CDS is in yellow, introns are in light blue, UTR is in pink, and intergenic regions are orange. **(C)** Heatmap with D’ estimates.

**FIGURE 9:**
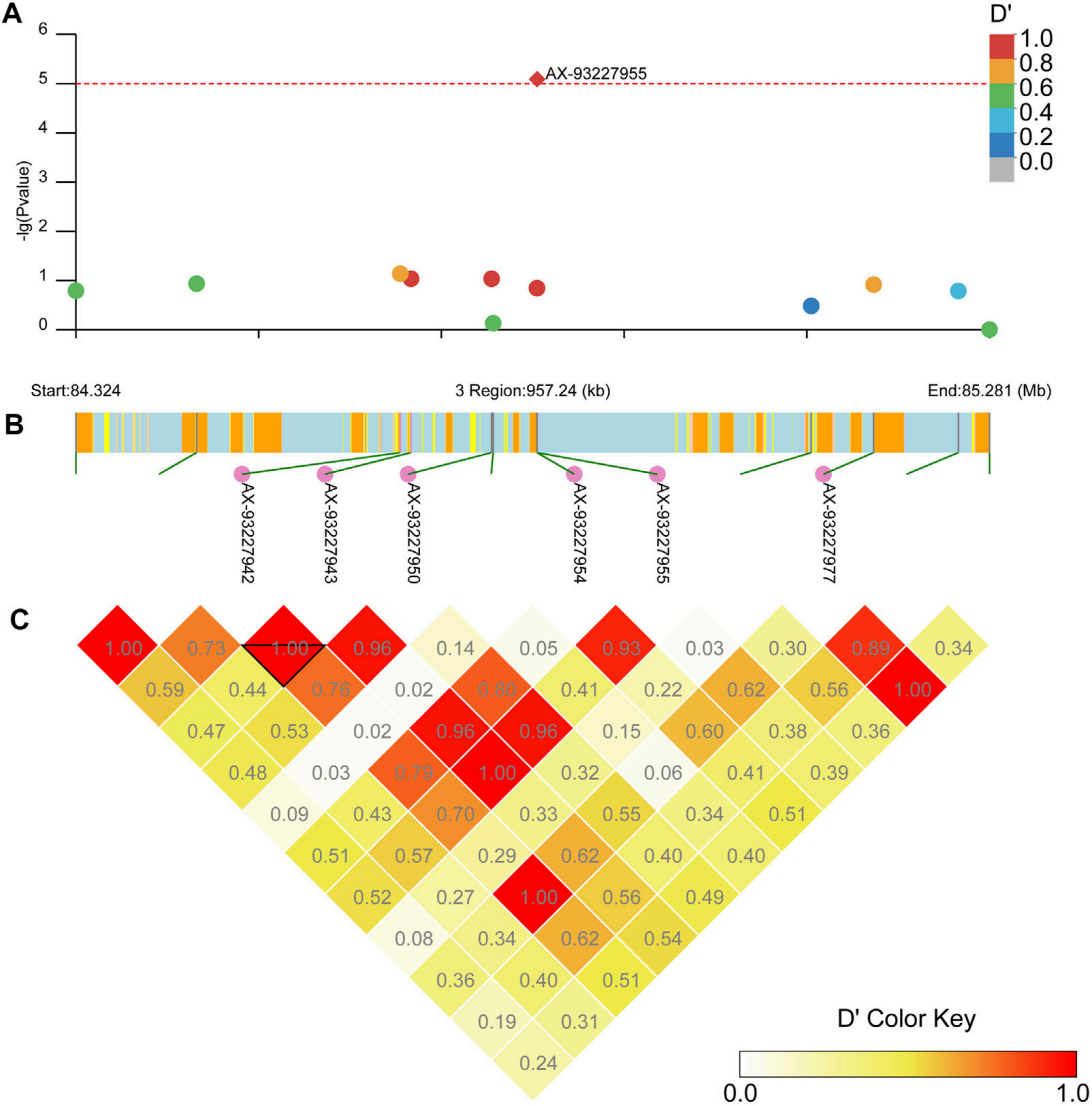
Linkage block analysis of loci AX-93227955 on *Ae. aegypti* chromosome 3 using the package LDBlockShow. **(A)** Manhattan plot with significance line (red). SNPs are colored following the key at top right. **(B)** Genes in the region where the CDS is in yellow, introns are in light blue, UTR is in pink, and intergenic regions are in orange. Loci linked to AX-93227955 are annotated. **(C)** Heatmap with D’ estimates.

Our LD block analysis showed no other SNPs linked to AX-93253438 on chromosome 2 (Figure 8). AX-93253438 is on the first intron of the gene AAEL011338, a protein-coding gene with three transcripts. The gene is annotated as an ankyrin repeat domain-containing protein at Vectorbase (Giraldo-Calderon et al., 2015). The other locus, AX-93227955, is in chromosome 3 and in linkage disequilibrium with multiple loci (*D’* > 0.8), revealing a presence of three large linkage blocks in the region (Figure 9). These three blocks span locus AX-93227942 at chr3: 84,663,866 bp to the locus AX-93227977 at chr3:85,159,865, with a total combined length of 495,999 bp. Several genes exist in this genomic region, but the locus AX-93227955 is on the first intron of the single transcript gene AAEL003896, a protein-coding gene annotated as a serine/threonine-protein kinase. The other genes within these LD blocks are: AAEL003893 (DNA repair protein), AAEL019783 (unknown function), AAEL017418 (unknown function), AAEL003868 (DNA repair protein), AAEL019783 (serine/threonine-protein kinase), AAEL003867 (unknown function), AAEL003891 (CTL transporter), AAEL003901 (unknown function), AAEL003887 (vacuolar membrane protein pep11), AAEL003874 (unknown function), and AAEL003872 (translationally-controlled tumor protein homolog—TCTP). This genomic region is approximately 2 Mb away from the *vgsc*, where the *kdr* known mutations are located (Matthews et al., 2018).

Our sliding window Fst estimates revealed genomic regions differentiated among the susceptible and resistant mosquitoes but not associated with pyrethroid resistance (Supplementary Figure S4). The global Weir and Cockerham weighted Fst estimate between Oiapoque and Macapa is 0.08, revealing low genetic differentiation. The comparison between resistant and susceptible within each population shows even lower genetic differentiation. For Macapa, the global Weir and Cockerham weighted Fst estimate is 0.003, while for Oiapoque, it is 0.01, revealing a slightly higher genetic heterogeneity within the Oiapoque population.

Our epistasis analysis revealed that the locus AX-93237274 on chromosome 2:133,109,990 is affected by the locus AX-93252255 on chromosome 3:300,181,334 (Supplementary Table S6). The AX-93237274 is approximately 40.34 Mb away from the other locus associated with pyrethroid resistance on chromosome 2 (locus AX.93253438). The nearest upstream gene to AX-93237274, 23,919 bp away, is AAEL012412, a gene with four transcripts coding for a slip protein. The downstream gene, 8,457 bp, is AAEL006568, which is a serine protease gene with only one transcript.

**FIGURE 10:**
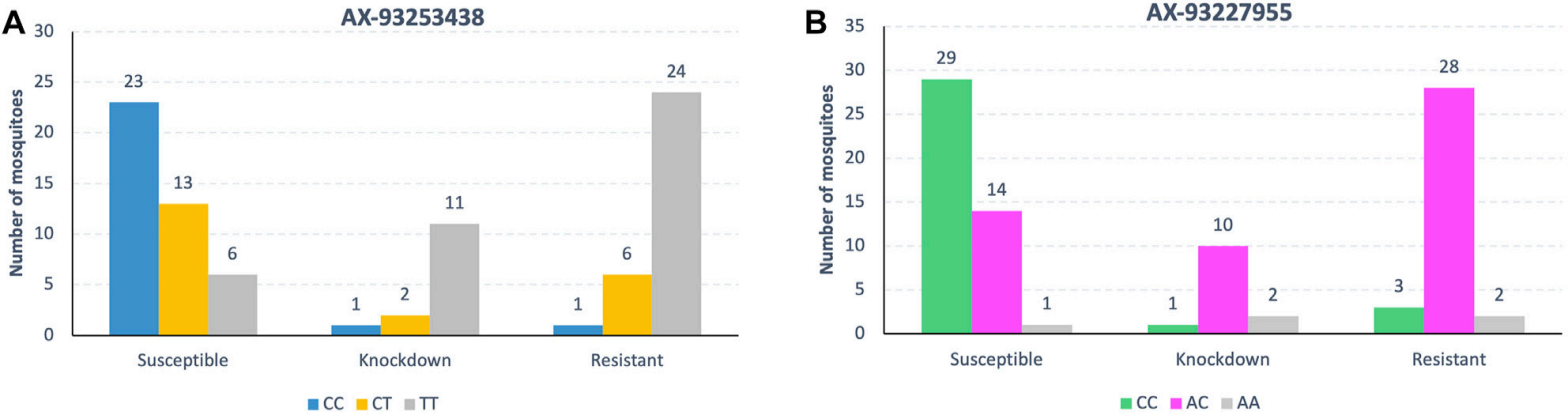
Genotype frequencies of the two loci associated with pyrethroid resistance in both *Ae. aegypti* populations. Mosquitoes with locus AX-93253438 **(A)** homozygous for the T allele display high resistance levels, while mosquitoes the locus AX-93227955 **(B)** heterozygous have the highest resistance levels.

Most resistant mosquitoes across both populations were homozygous “TT” at locus AX-93253438 and heterozygous “AC” at locus AX-93252255 (Figure 10). However, compound genotype frequency of loci AX-93253438 plus AX-93227955 revealed that the genotype CC/AC was most common in resistant/knockdown mosquitoes (Supplementary Table S7, and Figures S5, S6). The genotype TT/AC was twice as standard in susceptible mosquitoes than in resistant mosquitoes. Similarly, when we looked for the most frequent genotypes in resistant/knockdown mosquitoes, according to the *kdr* alleles, we found that the genotype CC/AC is typical in mosquitoes carrying *kdr* alleles R1R2 and R2R2 (Supplementary Table S8 and Figure S6). Most mosquitoes with the genotype CC at the locus AX-93253438 have the genotype AC at locus AX-93227955, except for only one mosquito (Supplementary Figure S5).

**FIGURE 11:**
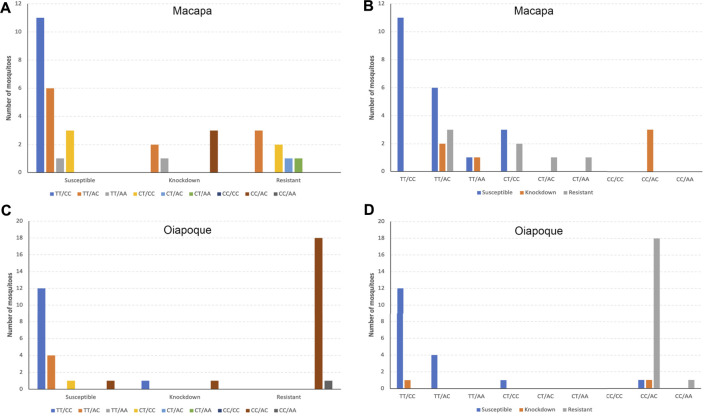
Compound genotypes frequencies of loci AX-9325343 + AX-93227955 according to each phenotype by population. **(A)** and **(B)** the compound genotype frequency of both loci in Macapa; **(C)** and **(D)** the compound genotype frequency of both loci in Oiapoque.

The genotype CC/AC (combined loci AX-9325343 + AX-93227955) was more frequent among resistant mosquitoes from Oiapoque and knockdown mosquitoes from Macapa. Most susceptible mosquitoes from both populations were TT/CC, followed by TT/AC (Figure 11).

**FIGURE 12:**
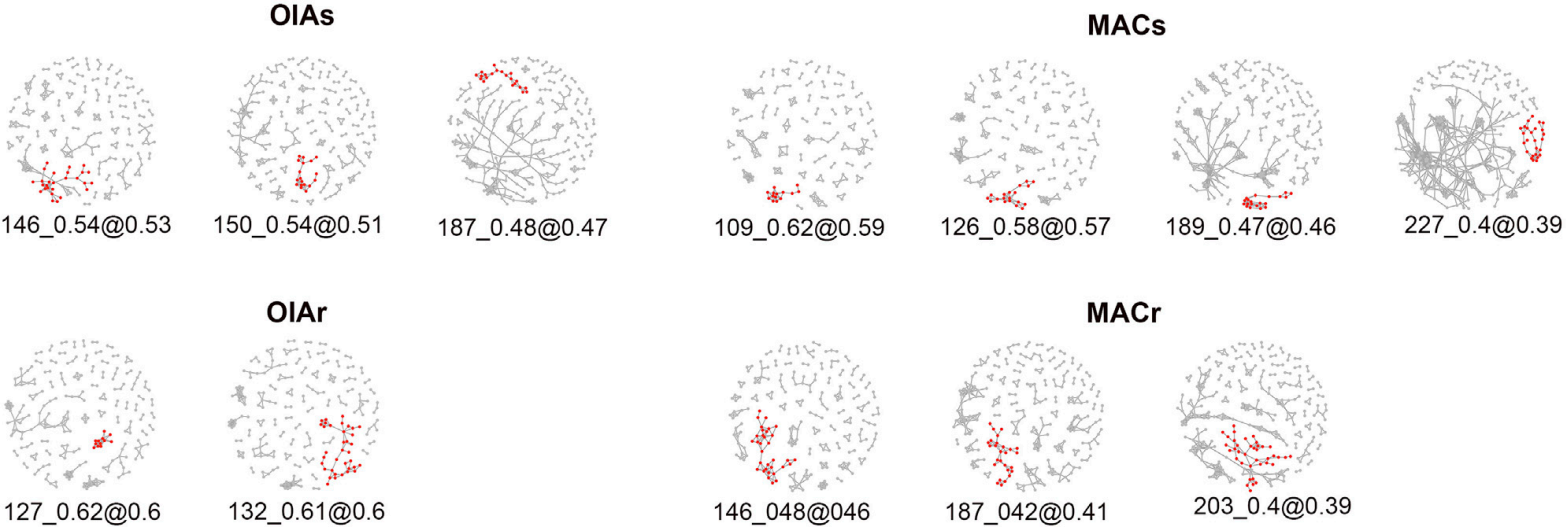
A snapshot of the entire networks at an LD threshold value just above that any single outlier clusters (SOCs) merge for resistant and susceptible individuals within each population. Each SOC is shown at an LD threshold where it is joined by a single link to other loci, in increasing order of threshold from right to left for each group. The compound outliers are not shown.

The linkage network analysis on chromosome 3 revealed distinguished linkage patterns between the resistant and susceptible individuals within both populations. After testing several parameters, from less to more stringent for cluster identification, we selected *φ* = 2 and the |*E*|_min_ = 40 which produced consistent and similar clustering patterns across all four groups (susceptible and resistant mosquitoes in both populations). In other words, the clusters we observed were reliably identified with less strict settings but plateau at the selected thresholds (Figure 12 and Supplementary Figure S8). The number of loci forming each single outlier cluster varied from 12 to 36, while the number of edges (nE) connecting these loci varied from 40 to 77 (Supplementary Table S10).

**FIGURE 13:**
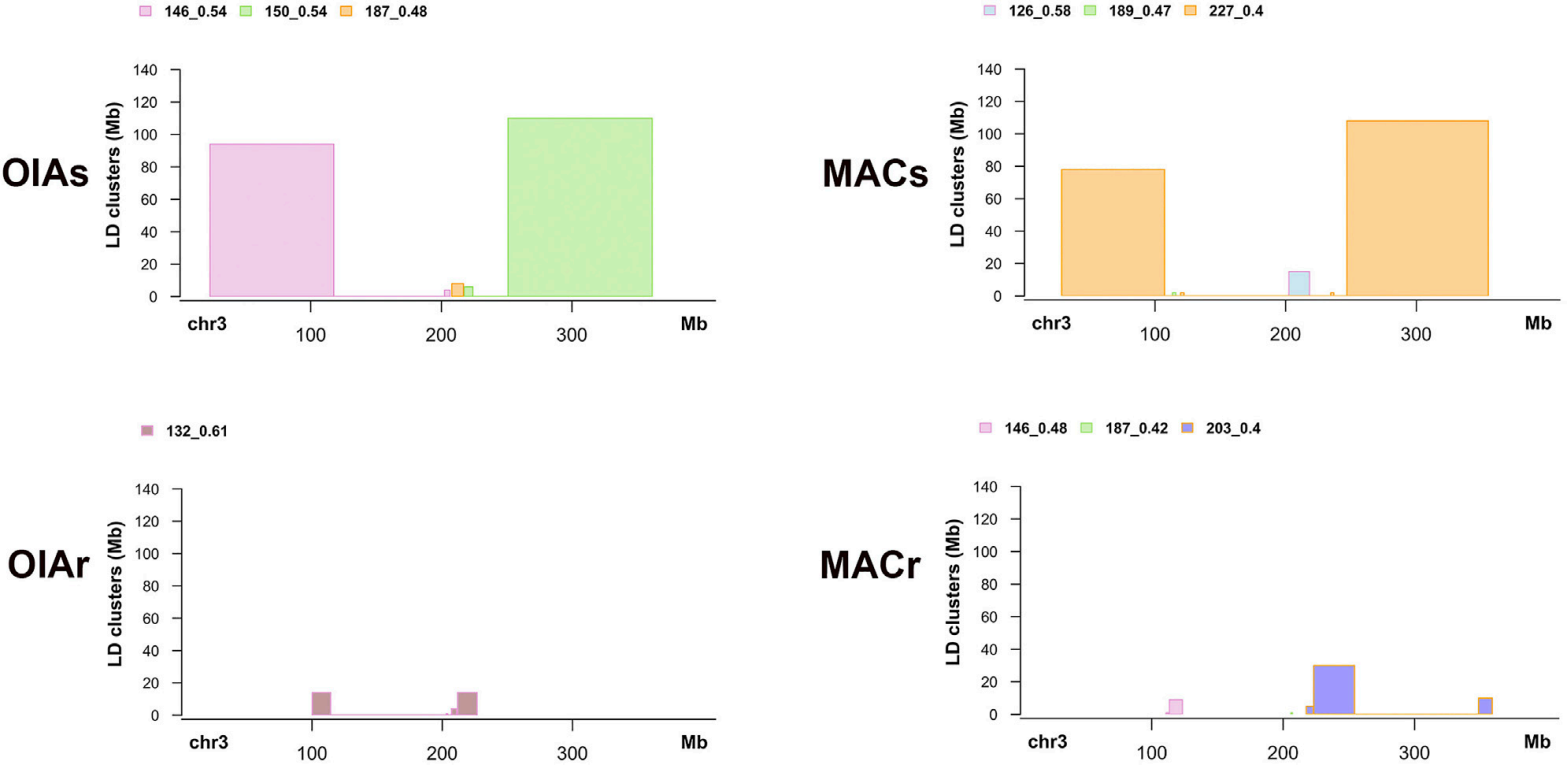
LD clusters bigger than 1 Mb in susceptible and resistant individuals within each population.

We extracted the list of SNPs for each SOC identified by LDna, obtained their genomic coordinates, and estimated the size of each cluster along chromosome 3. Due to the mosaic-like LD patterns, where a SOC may be compromised of larger and smaller LD blocks along the chromosome, we filtered out small blocks and plotted blocks bigger than 1 Mb (Figure 13 and Supplementary Table S11). The linkage patterns on susceptible individuals (OIAs and MACs) are similar across both populations, with two main LD clusters at relatively similar locations (Figure 13), ranging from 78 up to 110 Mb (Supplementary Table S11). There are also small LD blocks near the centromeric region in all individuals. However, we did not find large LD blocks within the resistant individuals (OIAr and MACr) in both population (Figure 13). The large LD block on the susceptible individuals includes the *vgsc* gene on chromosome 3, which is 110 and 108 Mb for OAIs and MACs, respectively.

## Discussion

Our genome-wide association analysis revealed two loci with strong association with resistance to pyrethroid in *Ae. aegypti*. Both linear regression in Plink and a mixed linear model in CGTA identified AX-93253438 (position in annotated genome in VectorBase) located on chromosome 2 and AX-93227955 on chromosome 3. The former is not strongly linked to nearby loci whereas the latter is in a large linkage block (Figures 8, 9).

While many GWAS studies employ many more markers than we have after quality control, for *Ae. aegypti* this is not a great concern. The power of GWAS is dependent on the number of markers per recombination unit. The *Ae. aegypti* genome has a low per mega base recombination rate (approximately 0.3 cM/Mb), with long recombination deserts (Juneja et al., 2014; Dudchenko et al., 2017; Chen et al., 2021), and our markers density after QC is on average about 12 SNPs/Mb, indicating good genomic coverage. Of course, recombination is not uniform across genomes, nevertheless, the number of markers we use have reasonably good power to detect significant associations.

### Genes Near GWAS Loci Associated Pyrethroid Resistance

The position of AX-93253438 is within an annotated gene (AAEL011338) implying it may function in metabolizing pyrethroids. It could be an important new target to study insecticide resistance in mosquitoes. This gene’s predicted protein has ankyrin repeats, one of the most common protein-protein interaction motifs known (Bork 1993). They are widely present in eukaryotes, and they can perform several functions from acting as transcriptional initiators, structural proteins, ion transporters, and signal transducers (Li et al., 2006; Voronin and Kiseleva 2007). In *Drosophila*, for example, mutations in *ankyrin 2* lead to the disintegration of the synaptic microtubule cytoskeleton, showing it is essential for synaptic stability (Koch et al., 2008). A previous study using exome capture enrichment in pyrethroid susceptible and resistant *Ae. aegypti* mosquitoes from Mexico have also identified enrichment for genes coding for proteins with ankyrin-domain in resistant mosquitoes (Campbell et al., 2019). Although AAEL011338 was not enriched in the Mexican mosquitoes, it reinforces an essential role of these gene families in pyrethroid resistance. In a pyrethroid-resistant population of *Anopheles funestus* from Senegal, one of the most overexpressed resistance-associated genes was also an ankyrin repeat domain protein (Afun005545) (Samb et al., 2016). Similarly, in the *Anopheles gambiae* resistant population to multiple insecticides from Burkina Faso, an ankyrin gene (*unc44*) was significantly overexpressed compared to susceptible mosquitoes (Kwiatkowska et al., 2013).

In *Ae. aegypti,* AAEL011338 has three transcripts, and their specific function and role in insecticide resistance remain unclear. Genes encoding ankyrin proteins in *Ae. aegypti* may play an essential role in enhancing pyrethroid resistance, especially AAEL011338 in our populations. Still, other ankyrin genes may also be involved as indicated by the resistant mosquitoes from Mexico exhibiting enrichment for different ankyrin genes (Campbell et al., 2019; Saavedra-Rodriguez et al., 2021). Since ankyrin is essential in microtubule dynamics (Zhu et al., 2020) and synaptic stability (Koch et al., 2008) in *Drosophila*, they could also be more directly implicated in resistance levels *via* structural changes in the synapse stability and neural activities.

The summary of gene expression experiments of VectorBase (Giraldo-Calderon et al., 2015) did not indicate that AAEL011338 has higher expression levels in pyrethroid-resistant mosquitoes. Its expression is slightly higher in some populations but was obscured by other genes and not identified as a significant player in metabolic resistance in a study comparing samples collected worldwide (Faucon et al., 2017). However, a resistant population from Thailand displayed increased mRNA levels compared to other populations from the United States, French Guiana, and French Polynesia, but only females were tested (Faucon et al., 2017).

The other locus associated with pyrethroid resistance in our study is AX-93227955 and is in the first intron of the single transcript gene AAEL003896, a protein-coding gene annotated as a serine/threonine-protein kinase with only one transcript. Protein-serine/threonine kinases participate in signal transduction cascades regulating several cellular processes including apoptosis, and cancerous processes (Roskoski 2010). In the cockroach *Periplaneta americana*, inhibition of serine/threonine phosphatase by cantharidin leads to toxicity and can be used for cockroach control (Sun et al., 2020). While phosphatase removes a phosphate group from a protein, kinases attach a phosphate group (Cheng et al., 2011). Therefore, AAEL003896 kinase could be adding a phosphate group activating vital proteins for pyrethroid resistance.

The gene expression profile of AAEL003896 summarized in Vectorbase (Giraldo-Calderon et al., 2015) indicated no significant differences between males and females (Dissanayake et al., 2010; Tomchaney et al., 2014), dengue virus infection (Behura et al., 2011), blood meal, exposure to pollutants (Poupardin et al., 2012), at developmental state, and *Wolbachia* infection (Kambris et al., 2009; Caragata et al., 2011). However, its expression was higher in a pyrethroid-resistant strains from Thailand (Faucon et al., 2017).

The locus AX-93227955 is in linkage disequilibrium with several other loci on chromosome 3 and is about 2 Mb away from the *vgsc, the* gene that contains *kdr* (Figure 9). There are several genes in this genome region, most with unknown functions. Other studies also pointed to several candidate genes involved in metabolic resistance or structural changes in the cuticle that could be correlated with increased resistance levels (Campbell et al., 2019; Vera-Maloof et al., 2020; Saavedra-Rodriguez et al., 2021). All these genes may have an additive or synergistic effect with the *kdr* alleles on the overall resistance of a specific population, and studies relying only on one population may arrive at a large list of genes that may not be directly linked to pyrethroid resistance but rather reflect local adaptation or temporal variations. The rise and spread of mutations in *kdr* alleles may occur independently (Cosme et al., 2020; Fan et al., 2020), with the fitness cost in different physiological aspects of *Ae. aegypti* to maintain such mutations (Brito et al., 2018; Smith et al., 2021) may reinforce differences existent in recent studies. The loss of *kdr* alleles is strain or population-dependent (Vera-Maloof et al., 2020), persisting for years after stopping use of pyrethroids (Macoris et al., 2018).

Our epistasis analysis indicated that AX-93227955 on chromosome 3 may interact with AX-93237274 on chromosome 2 (Supplementary Table S6). The affected locus is flanked by two protein-coding genes: AAEL012412 and AAEL006568. AAEL012412 product is a slip protein, which are proteins playing an essential role in cell migration, axon guidance during development, are upregulated in eukaryotes after nerve injury, maturation of neurons, and vascularization (Tong et al., 2019; Gonda et al., 2020). AAEL006568 codes for a serine protease, and it is not known what role it could play in insecticide resistance. One-third of all proteases are serine proteases, and they are involved in an exhaustive list of physiological processes (Hedstrom 2002).

### The GWAS Loci, *kdr* Alleles, and the Resistance Phenotypes

We genotyped all samples for *kdr* alleles using qPCR probes, comparing them to the phenotype after insecticide exposure plus the genotypes of the two loci we found in our GWAS. The genotype TT of the locus AX.93253438 on chromosome 2 is prevalent in knockdown and resistant mosquitoes and displays partial dominance (Veitia et al., 2018). The genotype AC of the locus AX-93227955 is ubiquitous in knockdown and resistant mosquitoes, with heterozygotes being more resistant than homozygotes, a case of overdominance (Figure 11). Most mosquitoes that were phenotypically knockdown/resistant were genotypically CC/AC (Supplementary Figure S5 and Table S7) for loci AX.93253438+AX-93227955. CC/AC is prevalent in mosquitoes carrying the *kdr* alleles R1R2 or R2R2 (Supplementary Figure S6 and Table S8), which are the *kdr* mutations known to confer the highest levels of pyrethroid resistance in Brazil (Melo Costa et al., 2020). It is unclear what mechanism caused such correlation, but these genotypes appear to act synergistically, increasing the resistance levels or lessening the cost of maintaining the *kdr* resistance alleles.

Our qPCR analysis revealed that only one mosquito genotyped with the SNP chip for GWAS did not have any *kdr* alleles (Figure 5). Most mosquitoes carry one or two *kdr* alleles, and the fitness cost of maintaining two co-occurring homozygous mutations in the absence of pyrethroid exposure is high: shorter average lifespan, smaller wings, longer larval developmental time, and fewer mosquitoes reaching the adult stage (Rigby et al., 2020). The few mosquitoes with genotype CC/AA or R1R2 that died from insecticide exposure may indicate complex interaction between the *kdr* alleles or simply be random.

### Differences Among Populations

The two populations used in our study were collected from cities less than 600 km apart and in the same climate zone in Northern Brazil. However, the resistance levels and frequency of *kdr* alleles were different. Oiapoque was considerably more resistant than Macapa (Figure 3), especially notable at high dosage of insecticide (Supplementary Table S3) and had a higher frequency of R1R2 and R2R2 *kdr* genotypes (Figure 4). Additionally, the most resistant compound genotype frequencies of loci AX-9325343 + AX-93227955 (CC/AC) are higher in Oiapoque than in Macapa. XIn contrast, in Macapa, the mosquitoes with knockdown resistance have the same genotype (CC/AC) (Figure 11). A caveat is the different dosages used in order to observe resistant and susceptible mosquitoes in Macapa and Oiapoque.

Although the cities are geographically close, they are connected by only one road. Oiapoque is well connected to other localities in French Guiana, for example, Saint-Georges, which are also connected to other localities in the Caribbean regions where the *kdr* allele R2 is present. In northern Brazil, the R2 allele is rare and probably originated from the Caribbean region (Salgueiro et al., 2019) due to cross-border gene flow. Our PCA (Figure 5) and Fst sliding-window analysis between susceptible and resistant phenotypes (Supplementary Figure S4) revealed clustering of samples; the genomic regions accounting for this are not the same genome regions differentiating susceptible and resistant mosquitoes. We controlled for population stratification in our GWAS and used multidimensional scaling to obtain covariates for our analysis. It is likely that the slightly different genetic background in these populations caused loci to be removed that would otherwise be significant due to local adaptation or drift.

### Linkage Patterns Could Indicate Assortative Matting Within Each Population

Recent studies have looked at the fitness cost of carrying the *kdr* mutations and other resistance mechanisms in *Ae. aegypti* and indicated that the cost of having such mutation depend on their allele frequencies and the genetic background of the natural population (Brito et al., 2013; Smith et al., 2016; Freeman et al., 2021; Rigby et al., 2021). Prominently, the reduction in male mating success due to reduction in the wing beat frequency in males (Rigby et al., 2021) could lead to assortative mating, where susceptible females may avoid resistant males.

Since both *kdr* alleles are present in both populations, we wanted to explore the effect of the introgression of the genomic regions on chromosome 3, where the *Na*_*V*_ gene is located. Although there are probes to genotype 11 nucleotide sites of the *Na*_*V*_ gene on the *Aegypti* SNP-chip, most were excluded due in our GWAS analysis due to low minor allele frequency. Mutations in the *Na*_*V*_ gene resulting in insecticide resistance are frequent in places where insecticides are continuously used. Therefore, we examined the effects of having *kdr* resistance alleles on the genome architecture of *Ae. aegytpi*. It is well-established that linkage between sites under selection will reduce the overall effectiveness of selection in finite populations (Hill and Robertson 1966) depending on their physical distance (Comeron et al., 2008). However, mosquitoes that carry insecticide resistance alleles have an enormous selective advantage where insecticides are used. Therefore, we want to compare the allele frequencies of loci on the chromosome 3 and estimate linkage disequilibrium (LD) in resistant and susceptible individuals. Previous studies of LD patterns in *Ae. aegypti* revealed relatively high levels of linkage across the genome, with *r* ^2^ max/2 (kb) up to 70 kb (Matthews et al., 2018). However, recent network analysis reveals mosaic-like LD patterns where large clusters of SNPs in high LD are interspersed with other smaller clusters, due to recombination breaking up linkage patterns (Li et al., 2018).

The LD network analysis revealed remarkably distinct and consistent patterns within susceptible and resistant mosquitoes in both populations (Figure 13). Since we used the same set of loci to estimate LD in all individuals, differences in their frequency lead to different *r* ^2^ estimates used in our analysis. It is an indication that the introgression of the genomic regions with the *kdr* alleles changed the linkage patterns within each population in a similar manner in both localities.

The effect of *kdr* introgression has also been studied in *Anopheles gambiae* and *Anopheles colluzii* in Africa (Norris et al., 2015). This study indicated that selection pressure from pyrethroid treated bed nets changed the fitness landscape favoring the transfer of resistance alleles by hybridization. Heavy insecticide use favored the survival of hybrids between the species and produced selection pressure sweeping the genomic region where the *kdr* mutations cross species boundaries. In this case, the *An. coluzzii* population was susceptible before the introgression of the *kdr* alleles. However, there is no evidence of assortative mating due to insecticide pressure and the presence of *kdr* alleles. In our study, the *Ae. aegypti* populations from Oiapoque and Macapa have different *kdr* haplotypes (Cosme et al., 2020) and different levels of pyrethroid resistance. When mosquitoes from both populations were exposed to the lower dose (600 mg/L), all mosquitoes from Oiapoque were resistant compared to 25% in Macapa. When we doubled the dose (1,200 mg/L), 75% of the mosquitoes from Oiapoque still resistant (Figure 3). However, the susceptible and resistant mosquitoes display different LD patterns in the chromosome with the *kdr* mutation, indicating that the introgression of the genomic regions with the *kdr* mutations changed the chromosome architecture independent of the genetic background (Figure 13).X.

The *kdr* mutations in *Ae. aegypti* can lead to assortative matting caused by a significant reduction male matting success (approximately 17%) due reduction in wing beating frequency in pyrethroid-resistant males in laboratory conditions (Rigby et al., 2021). The changes in LD on chromosome 3 that we see between susceptible and resistant mosquitoes could be due to assortative matting after the introgression of the genomic regions with the *kdr* mutations. There is evidence that these Northern populations re-invaded Brazil from Venezuela or other neighboring country after the eradication programs decades ago (Kotsakiozi et al., 2017). The local adaptation to Amazon environments may have led to the linkage patterns we observed on chromosome 3 in susceptible mosquitoes of both populations. However, with the introgression of the *kdr* mutations, and its enormous selective advantage, the linkage patterns were broken by recombination. However, due to reduction in male matting success because of the fitness cost of these mutations, assortative matting may be occurring at a higher rate than observed in laboratory conditions. Finally, effective population size estimates for *Ae. aegypti* populations indicate relative low *Ne*, a few hundreds and even lower in some populations (Saarman et al., 2017), potentially intensifying the effects of assortative matting.

## Conclusion

Our study revealed two new loci directly associated with pyrethroid resistance. The genotype frequencies of these loci correlate with the *kdr* genotypes in resistant and susceptible mosquitoes, especially in Oiapoque. We also observed significant differences between the populations in deltamethrin LC50 and frequency of *kdr* alleles. Although the allele frequencies of *kdr* alleles will probably decrease over time in the absence of pyrethroid use, it will be of interest to follow the other loci identified here that are not part of *vgsc.* We do not know if these loci impose a negative fitness cost in the absence of pyrethroid insecticides. Our linkage network analysis indicate reduction in linkage in chromosome 3 within resistant and susceptible mosquitoes from both populations. This is possibly due to assortative matting after the introgression of the *kdr* mutations and reduction of selective pressure in the absence of pyrethroid insecticides.

## Data Availability

The datasets presented in this study can be found in online repositories. The names of the repository/repositories and accession number(s) can be found below: https://datadryad.org/stash, QdrmeaPpiMmeuIxKxyfGSUNetGkL4uGn3HYRLPAiNbs.
