# Supplementary figures and images for: Genome-wide Association Study Reveals New Loci Associated With Pyrethroid Resistance in Aedes aegypti
Source: Front Genet. 2022 Apr 11;13:867231. doi: 10.3389/fgene.2022.867231 (PMC9035894; doi:10.3389/fgene.2022.867231)

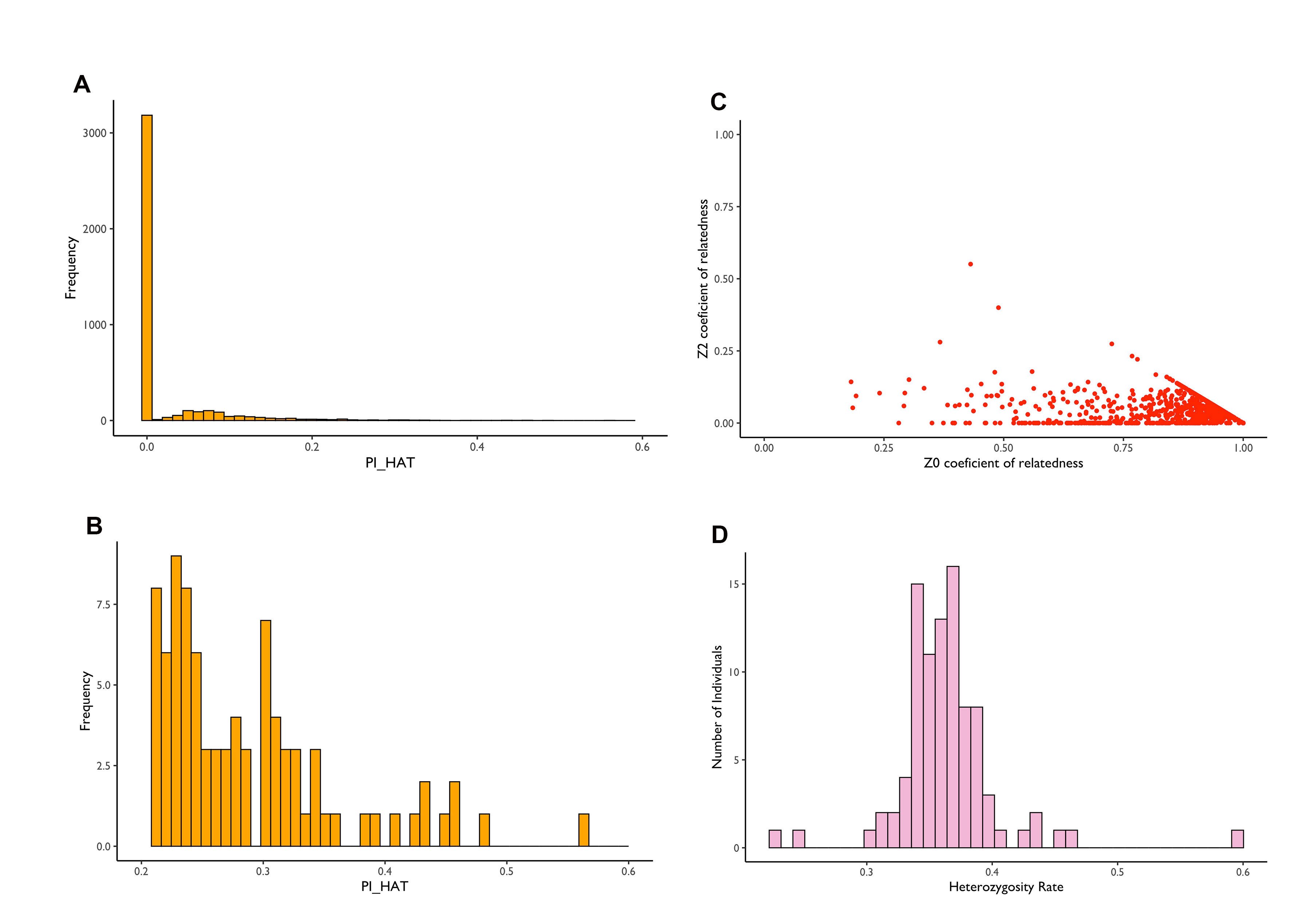

Supplement: Supplementary file 1 [file Image3.JPEG]

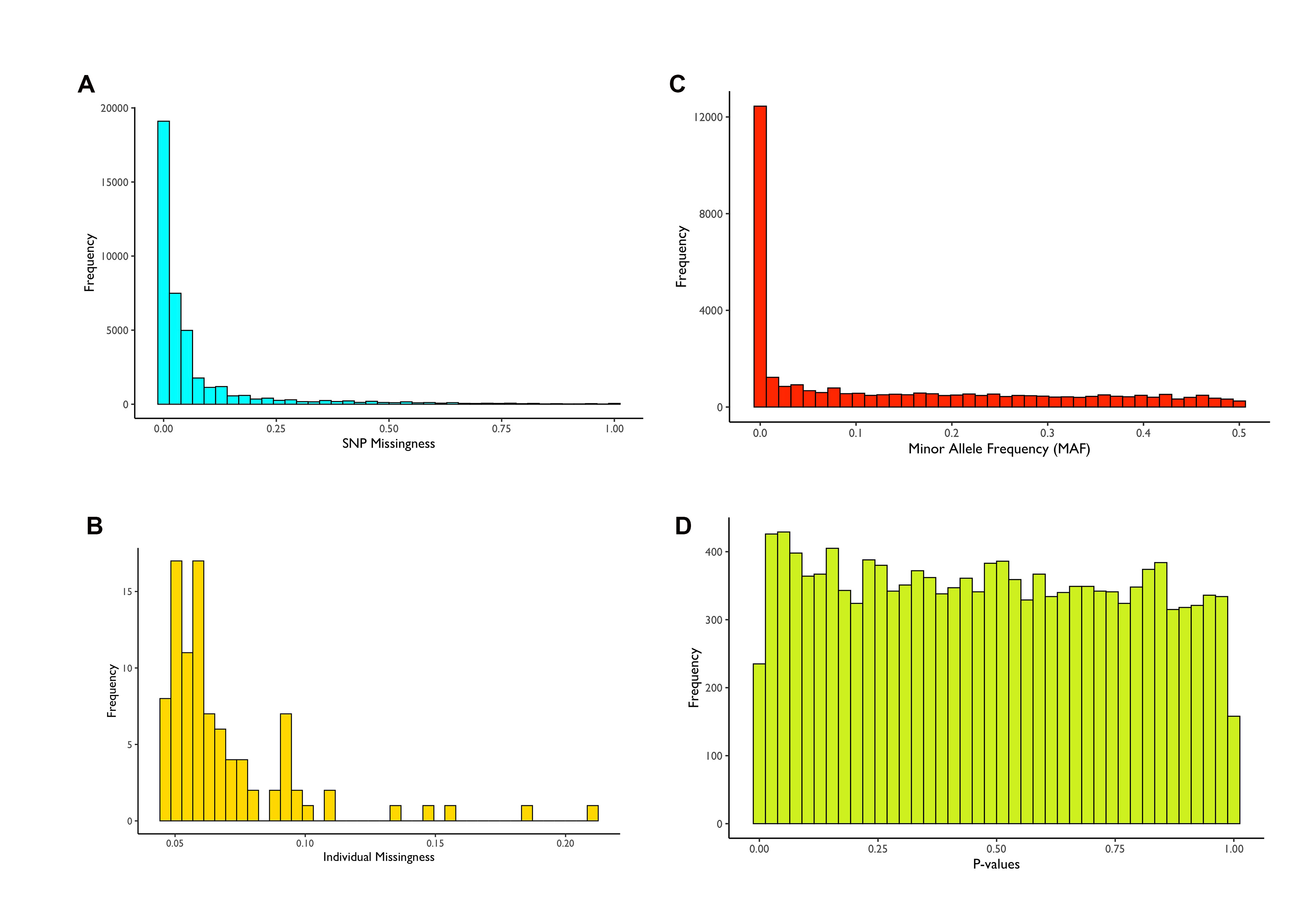

Supplement: Supplementary file 3 [file Image1.JPEG]

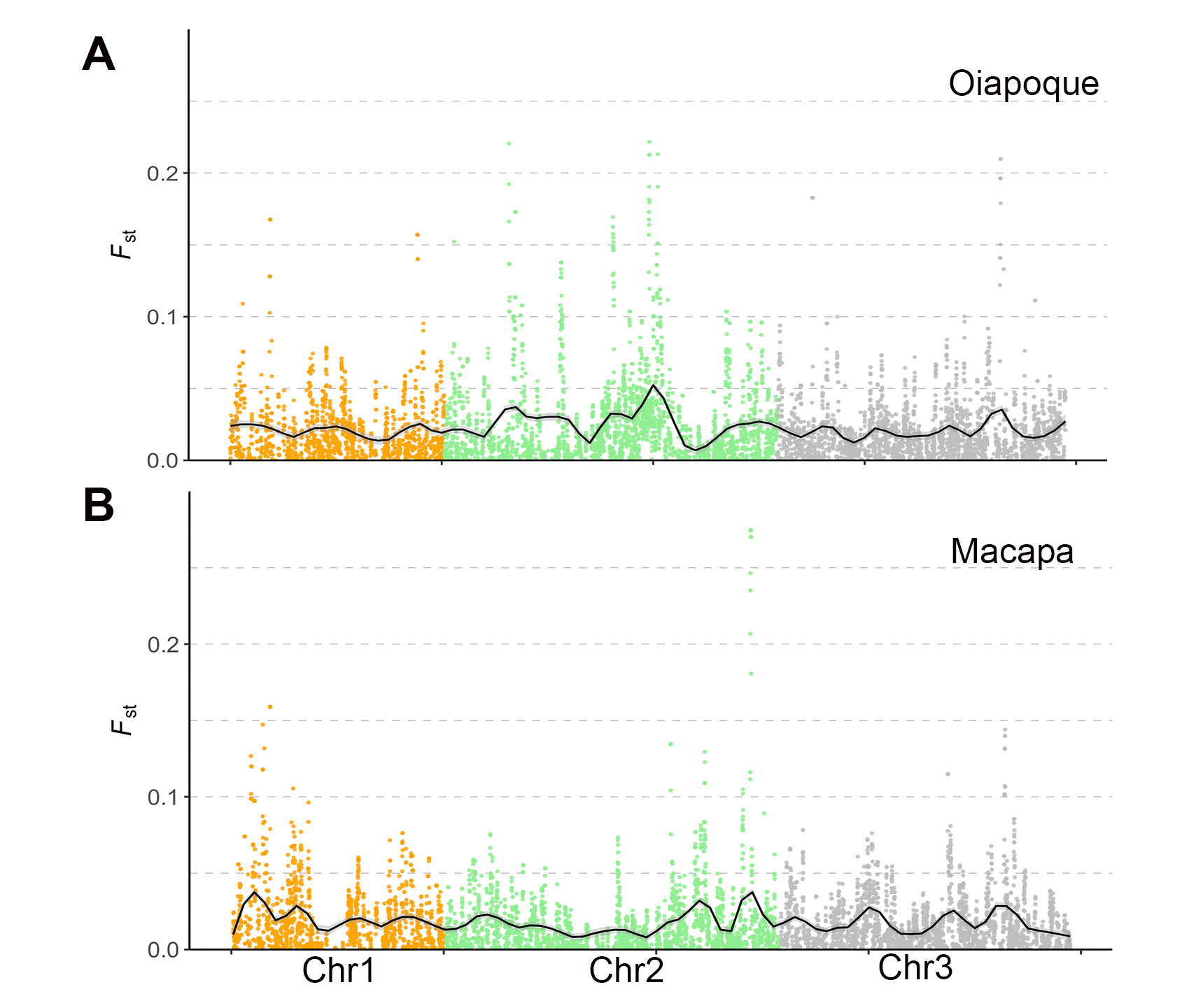

Supplement: Supplementary file 4 [file Image4.JPEG]

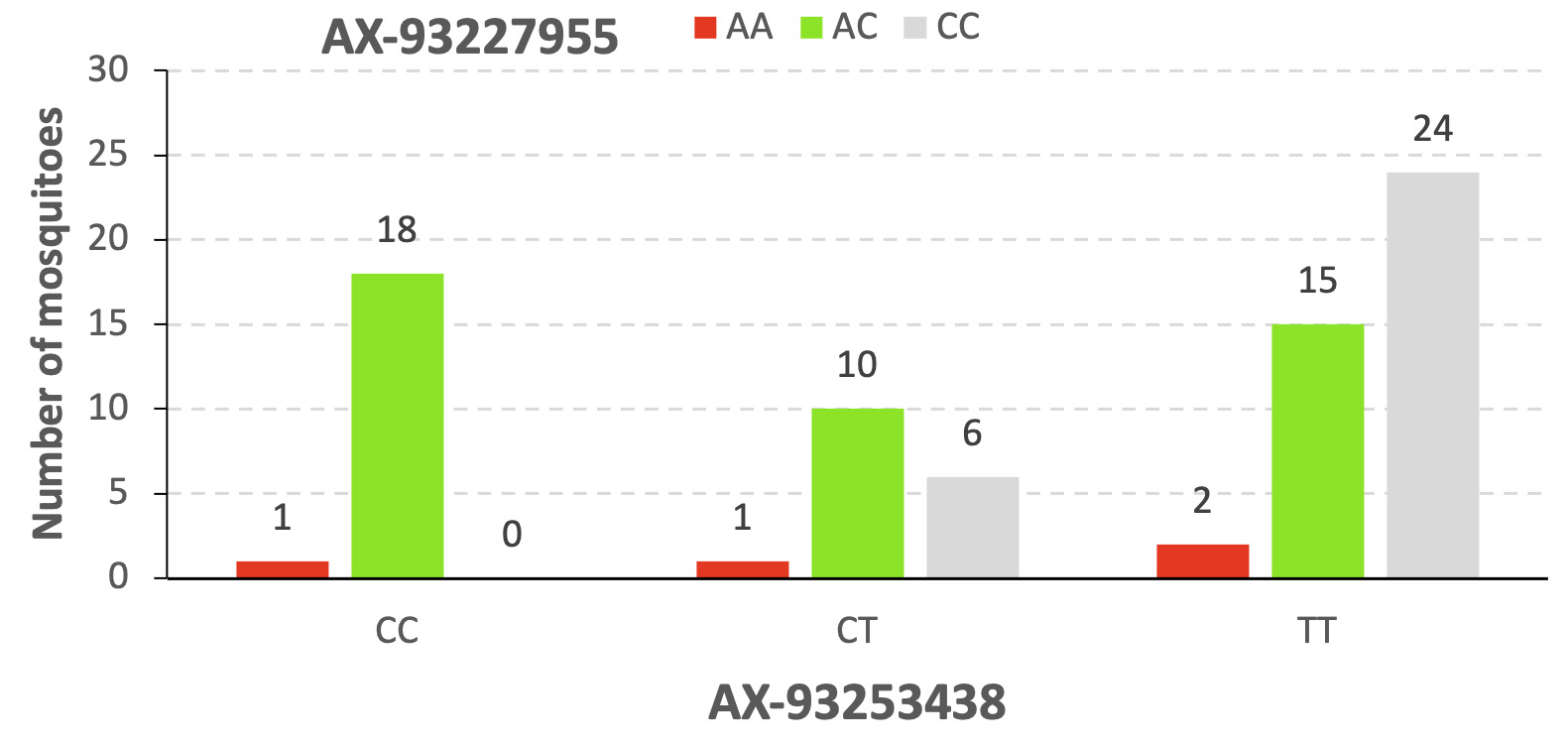

Supplement: Supplementary file 5 [file Image7.JPEG]

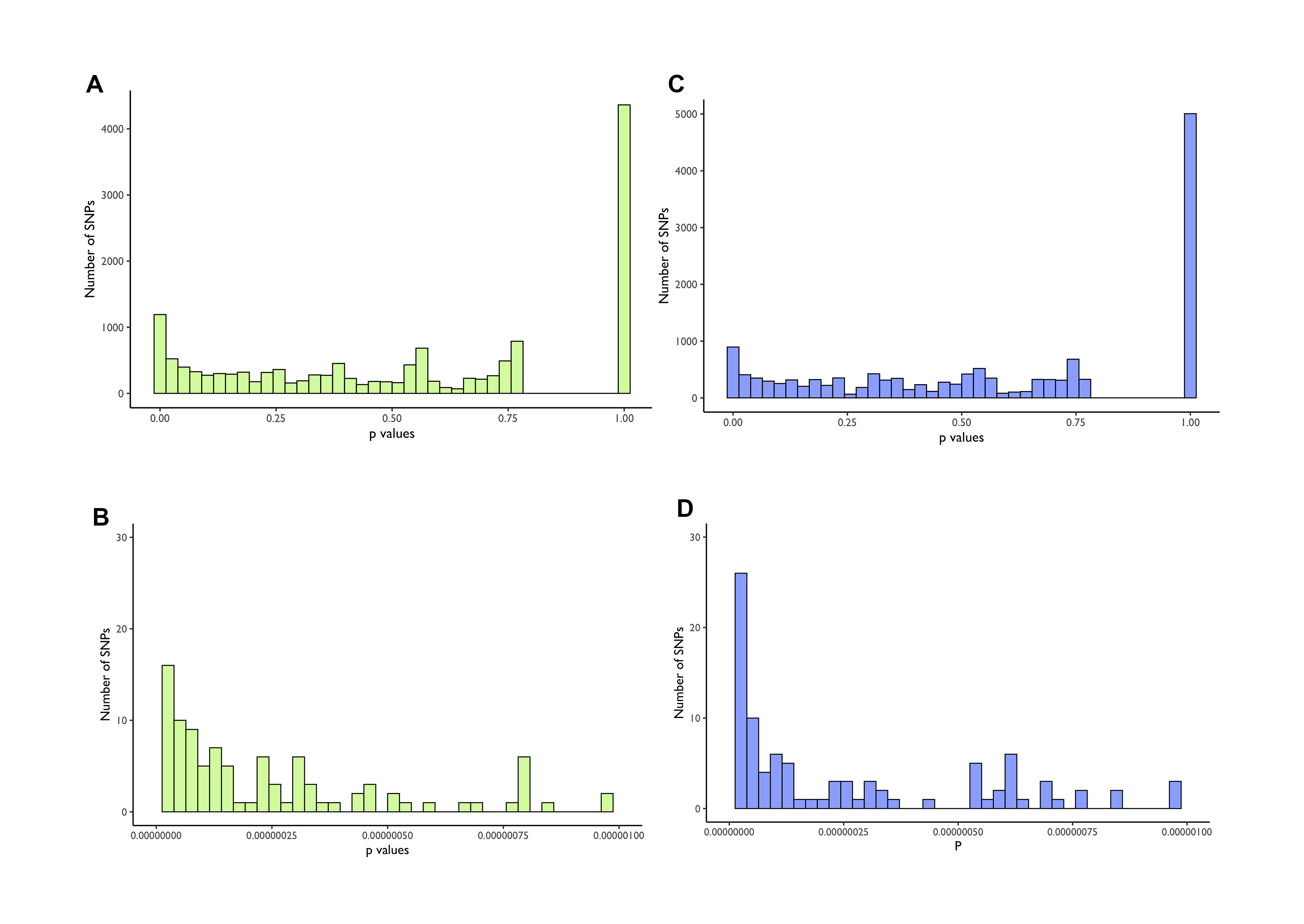

Supplement: Supplementary file 6 [file Image2.JPEG]

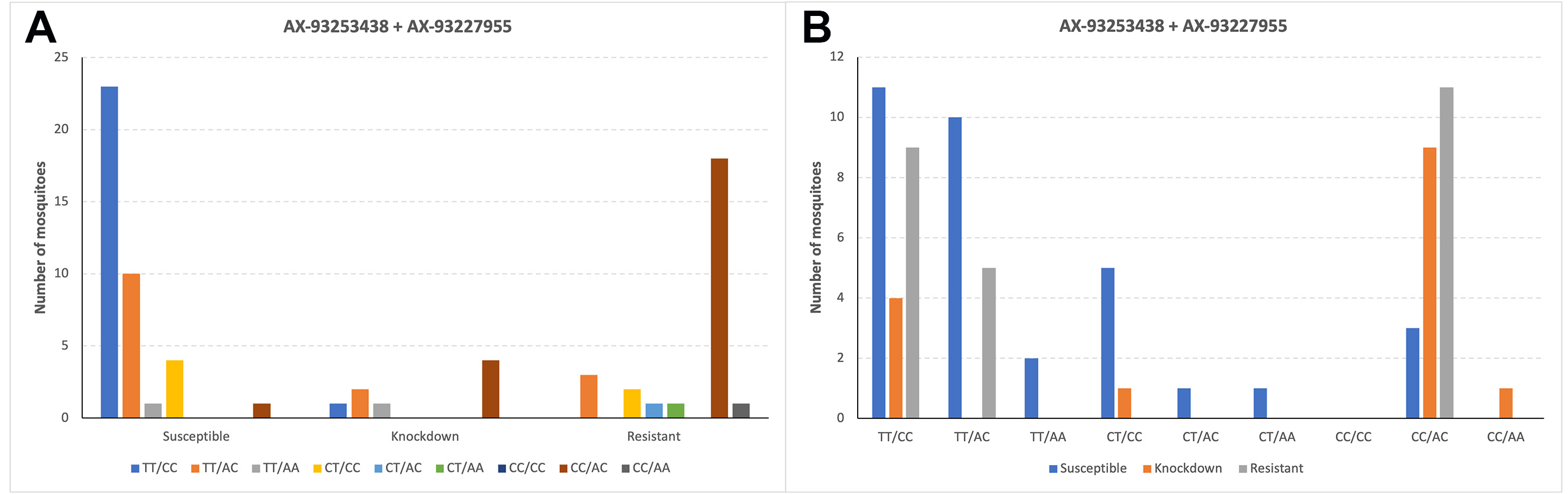

Supplement: Supplementary file 7 [file Image5.JPEG]

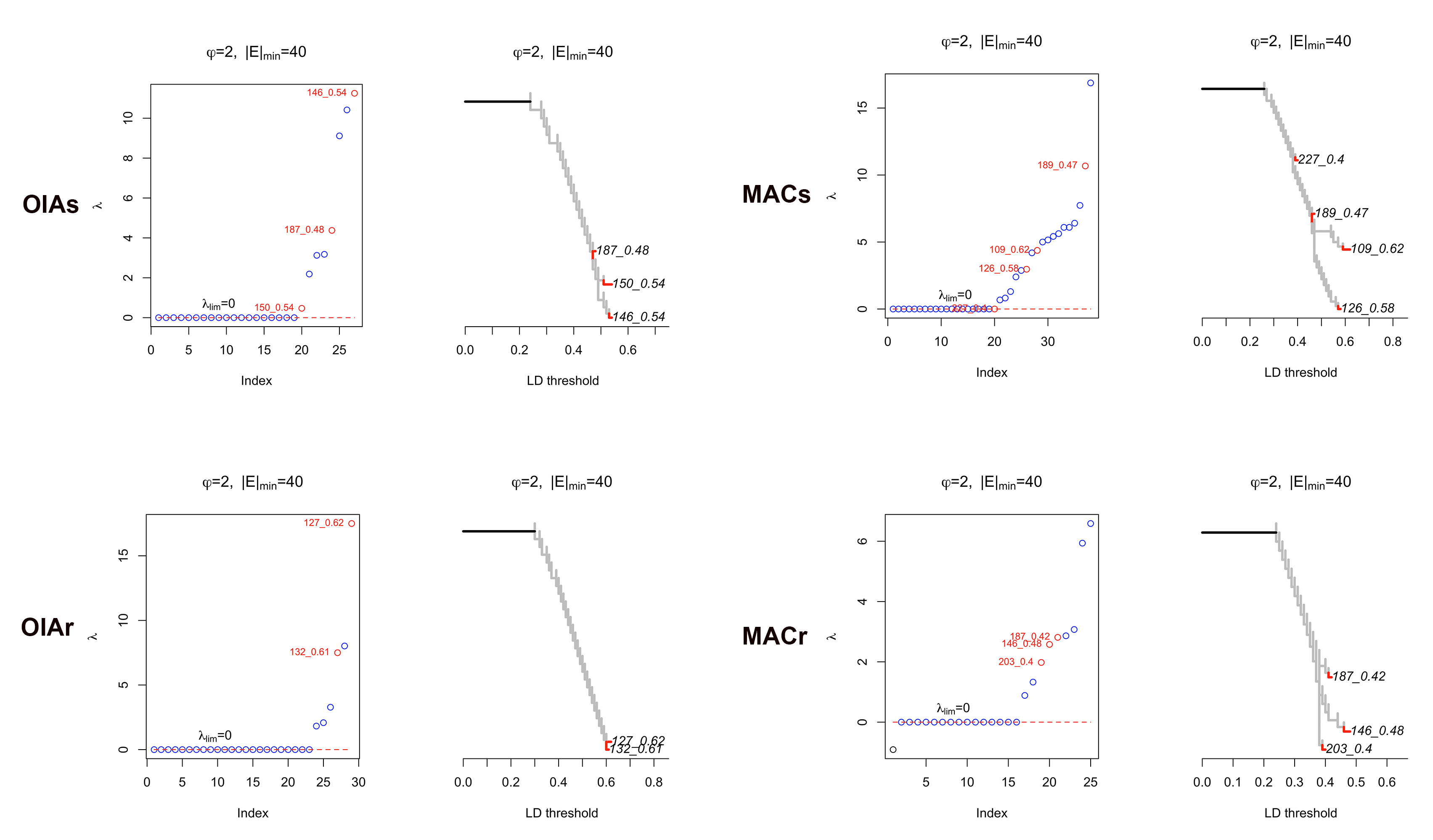

Supplement: Supplementary file 10 [file Image8.JPEG]

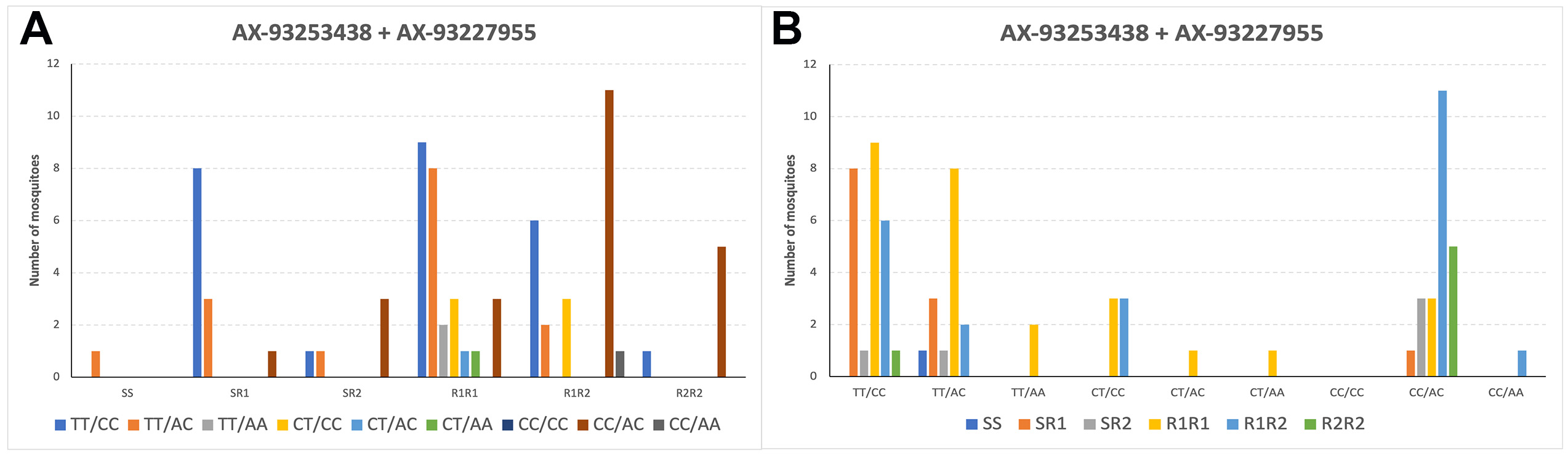

Supplement: Supplementary file 11 [file Image6.JPEG]
