# Supplementary material for: Genome-wide Association Study Reveals New Loci Associated With Pyrethroid Resistance in Aedes aegypti
Source: Front Genet. 2022 Apr 11;13:867231. doi: 10.3389/fgene.2022.867231 (PMC9035894; doi:10.3389/fgene.2022.867231)
Supplement: Supplementary file 2 [file DataSheet3.docx]

**List of Figures**

**Figure 1**. Location of our egg collection sites, Oiapoque and Macapa, Amapa, Brazil. Map,*Google Maps.*Accessed 9 June 2021.

**Figure 2.** Experimental design. We exposed mosquitoes from each population to deltamethrin, via impregnated papers in tubes, for one hour. We kept females that were alive in -80^o^C for DNA extraction. We stored the mosquitoes that were not moving for 24h to separated knockdown resistant mosquitoes from susceptible (dead). After 24h, we divided the mosquitoes that survived the exposure but were knocked down from the dead. We conducted three biological replicates. We extracted DNA from all mosquitoes simultaneously, genotyped them for the *kdr* resistance alleles by qPCR, and preceded with genotyping using the Axion *aegytpti1* SNP chip.

**Figure 3.** The proportion of the phenotypes within each population. Resistant (mosquitoes alive after 1-hour of the exposure), knockdown resistance (mosquitoes were knocked down 1h after exposure but were active 24 hours later), and susceptible (mosquitoes died after 1 hour of exposure and were not active 24h later). We genotyped 95 individuals for our GWAS. See Table 3 for more details.

**Figure 4.** The proportion of genotypes for each population. Two phenotypes: Resistant (R) – mosquitoes that did not die 24h after exposure, and Susceptible (S) – mosquitoes were dead 24h after the exposure. Mosquitoes with the genotypes SS, SR1, and SR2 are phenotypically susceptible. Mosquitoes with the genotypes R1R1, R1R2, and R2R2 are phenotypically resistant, but the resistance levels are different, with the R2R2 genotype giving the highest level of resistance. Mosquitoes were genotyped via qPCR using primer and probes described in Table 1.

**Figure 5.** Principal component analysis with all the mosquitoes used in our association analyses. Each mosquito was genotyped using qPCR to identify each *kdr* genotype they had.

**Figure 6.** GWAS in mosquitoes with different levels of knockdown resistance. A. Principal component analysis showing two different clusters; B. Quantile-quantile plot with confidence intervals; C. Manhattan plot with the SNPs with the lowest *p* values annotated per chromosome. Green dots are SNPs on the *vgsc* gene. The SNP chip has 11 SNPs on the *vgsc* gene; only 3 SNPs passed our filtering, with most being removed due to low minor allele frequency.

**Figure 7**. Genotype’s frequency (%) of the alleles associated with pyrethroid resistance in *Ae. aegytpi.* Most homozygous mosquitoes carrying the allele T, loci AX-93253438, and C, loci AX-93227955, died within one hour of exposure to deltamethrin.

**Figure 8.** Linkage block analysis of loci AX-93253438 on *Ae. aegypti* chromosome 2 using the R package LDBlockShow. A. Manhattan plot with significance line (red). SNPs are colored following the key at top right. B. Genes in the region with SNPs marked as green lines, where the CDS is in yellow, introns are in light blue, UTR is in pink, and intergenic regions are orange. C. Heatmap with D’ estimates.

**Figure 9.** Linkage block analysis of loci AX-93227955 on *Ae. aegypti* chromosome 3 using the package LDBlockShow. A. Manhattan plot with significance line (red). SNPs are colored following the key at top right. B. Genes in the region where the CDS is in yellow, introns are in light blue, UTR is in pink, and intergenic regions are in orange. Loci linked to AX-93227955 are annotated. C. Heatmap with D’ estimates.

**Figure 10.** Genotype frequencies of the two loci associated with pyrethroid resistance in both *Ae. aegypti* populations. Mosquitoes with locus AX-93253438 homozygous for the T allele display high resistance levels, while mosquitoes the locus AX-93227955 heterozygous have the highest resistance levels.

**Figure 11.** Compound genotypes frequencies of loci AX-9325343 + AX-93227955 according to each phenotype by population A and B. The compound genotype frequency of both loci in Macapa; C and D. The compound genotype frequency of both loci in Oiapoque.

**Figure 12.** A snapshot of the entire networks at an LD threshold value just above that any single outlier clusters (SOCs) merge for resistant and susceptible individuals within each population. Each SOC is shown at an LD threshold where it is joined by a single link to other loci, in increasing order of threshold from right to left for each group. The compound outliers are not shown.

**Figure 13.** LD clusters bigger than 1Mb in susceptible and resistant individuals within each population.

**Supplemental Figures**

**Supplemental Figure 1.** Quality control steps for SNP filtering before GWAS. A. SNP missingness – we removed SNPs that were not present in at least 90% of the mosquitoes; B. Individual missingness – we removed mosquitoes that did not retain at least 90% of the SNPs; C, minor allele frequency – we removed SNPs whose minor allele frequency was below 10%; D. Uncorrected *p* values distribution.

**Supplemental Figure 2.** Hardy-Weinberg equilibrium (HWE) filtering before the GWAS. A. Distribution of SNPs in Macapa; B. Zoom to show the SNPs removed due to small *p* values in Macapa population; C. Distribution of SNPs in Oiapoque; B. Zoom to show the SNPs that were removed due to small *p* values in Oiapoque population.

**Supplemental Figure 3.** Quality control steps before the GWAS. A. PI_HAT distribution using both populations; B. Zoom shows mosquitoes with PI_HAT above 0.2; C. Z0 and coefficients of relatedness; D. Heterozygosity distribution.

**Supplemental Figure 4.** Sliding window Fst estimates between resistant and susceptible mosquitoes from Oiapoque (A) and Macapa (B). Each point is the Weir and Cockerham weighted Fst estimate for a 1Mb window in the genome with 100kb steps.

**Supplemental Figure 5.** Compound genotypes frequencies of loci AX-9325343 + AX-93227955 after insecticide exposure. A. Genotype frequency according to the phenotypes; B. Phenotype frequency according to the compound genotypes of both loci. See Table 6 for more details.

**Supplemental Figure 6.** Compound genotypes frequencies of loci AX-9325343 + AX-93227955 according to their *kdr* genotype by qPCR after insecticide exposure. A. The compound genotype frequency of both loci according to the *kdr* genotype frequency done by qPCR; B. The *kdr* genotype frequency following the compound genotype frequency of our GWAS significant SNPs. See Table 7 for more details.

**Supplemental Figure 7.** Genotype frequencies of loci AX-9325343 and AX-93227955 in our study.

**Supplemental Figure 8.** LDna clusters formed at different LD thresholds using the same parameters for susceptible and resistant mosquitoes within each population. All λ values in increasing order with values above λ = 0 corresponding to outlier clusters. Parameter values for φ and |E|min are shown above plots. Single compound outliers are in red and compound outliers in blue.
