# Supplementary material for: Genome-wide Association Study Reveals New Loci Associated With Pyrethroid Resistance in Aedes aegypti
Source: Front Genet. 2022 Apr 11;13:867231. doi: 10.3389/fgene.2022.867231 (PMC9035894; doi:10.3389/fgene.2022.867231)
Supplement: Supplementary file 8 [file DataSheet1.PDF]

Statistical Power

We followed the GWAS course by Dr. Matti Pirinen from University of Helsinki (PIRINEN 2020) to perform our power analysis. In our GWAS, we used a significance threshold of 1e-05 (-LOG_{10} = 5 on Manhattan plot), and correction of multiple testing using Bonferroni correction $p < 0.05$ (BENJAMINI AND HOCHBERG 1995), and found two SNPs above the significance threshold. The significance testing with the multiple testing correction is the convenient method to summarize the evidence for association between a trait and genetic variant (SHAM AND PURCELL 2014). It is worth to remember that increased linkage increases the probability to find variants associated with a trait, and *Ae. aegypti* genome architecture indicates that the distance of half r^2 between 50kb and 120kb, varying from among populations.

Simulations (R code from Dr. Pirinen**):**
A. Distributions

```
n = 90 #individuals

p = 14000 #SNPs for both null and alternative

f = 0.1 #MAF

b.alt = 0.2 #effect size under the alternative hypothesis (we tested differen
t values)

x = rbinom(n, 2, f) #genotypes at 1 SNP for n ind

y = scale( rnorm(n) ) #random phenotype normalized to have sample sd=1

se = summary( lm( y ~ x ) )$coeff[2,2] #pick se, and assume it stays constant
and independent of beta

b.hat.null = rnorm(p, 0, se) #estimates under null

b.hat.alt = rnorm(p, b.alt, se)  #estimates under alternative

par(mfrow=c(1,2))

# Draw observed densities of z-scores

plot(NULL, xlim = c(-3,6), ylim = c(0,0.5), xlab = "z",

     ylab = "density", col = "white") #empty panel for plotting

lines(density( (b.hat.null/se) ), col = "black", lwd = 2) #Wald stat for null
variants

lines(density( (b.hat.alt/se) ), col = "red", lwd = 2) #Wald stat for alterna
tive variants

# add theoretical densities for z-scores

x.seq = seq(-3, 6, 0.01)

lines(x.seq, dnorm(x.seq, 0, 1), col = "blue", lty = 2) #for null

lines(x.seq, dnorm(x.seq, b.alt/se, 1), col = "orange", lty = 2) #for alterna
tive

# Draw observed densities of z^2

plot(NULL, xlim = c(0,35), ylim = c(0,1), xlab = expression(z^2),
```

```
    ylab = "density", col = "white") #empty panel for plotting

lines(density( (b.hat.null/se)^2 ), col = "black", lwd = 2) #chi-square stat for null variants

lines(density( (b.hat.alt/se)^2 ), col = "red", lwd = 2) #chi-square stat for alternative variants

# Let's add theoretical densities of the chi-square distributions

x.seq = seq(0, 35, 0.01)

lines(x.seq, dchisq(x.seq, df = 1, ncp = 0), col = "blue", lty = 2) #ncp=0 for null

lines(x.seq, dchisq(x.seq, df = 1, ncp = (b.alt/se)^2), col = "orange", lty = 2) #ncp = (beta/se)^2 for alternative

legend("topright", leg = c("NULL obs'd","ALT obs'd","NULL theor","ALT theor"),

       col = c("black","red","blue","orange"),

       lty = c(1,1,2,2), lwd = c(2,2,1,1) )

#Let's add significance thresholds corresponding to 0.05 and 5e-8

#By definition, the thresholds are always computed under the null.

q.thresh = qchisq( c(0.05, 5e-5), df = 1, ncp = 0, lower = FALSE)

abline(v = q.thresh, col = c("darkgreen", "springgreen"), lty = 3)

text( q.thresh+2, c(0.4,0.4), c("P<0.05","P<5e-8") )
```

B. Proportion of the distribution to the right of a given threshold value

```
q.thresh = qchisq(c(0.05,5e-5), df = 1, ncp = 0, lower = FALSE) #repeating thresholds in chi-square units

pchisq(q.thresh, df = 1, ncp = (b.alt/se)^2, lower = FALSE) #correspond to right tail probabilities
```

Results
1. Effect size of 0.2, MAF of 10%, and 14k SNPs

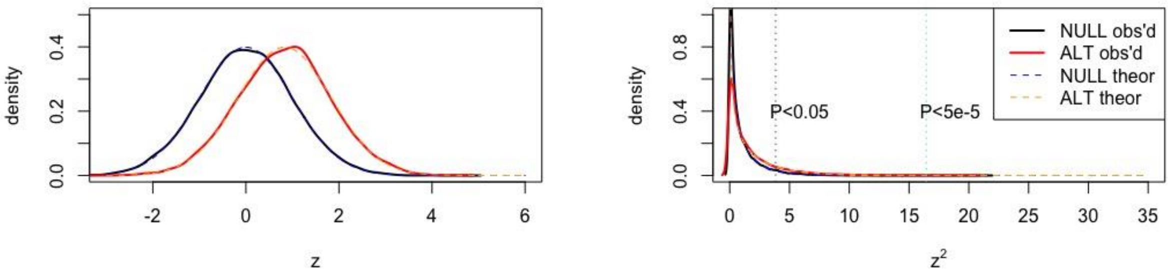

Interpretation of the distributions: the theoretical distributions (dotted lines) and observed ones match well. We see that most of the alternative distribution is partially to the left of the significance threshold of 0.05 but we have a tail at the left of the 5e-5. Therefore, we would discover some of the variants at the level 0.05 but almost none at the genome-wide significance level of 5e-5.

Interpretation of the proportions at the right of a given threshold*:* the significance threshold would be 0.1326402629 0.0006372471. Meaning we would have a probability of 13% to detect a variant at significance threshold of 0.05, when the effect size is 0.02 sd units for our quantitative phenotype, with MAF of 10% and sample size of 90 mosquitoes. We will also have a probability of 0.06% to detect a significant variant at genome-wide significance threshold of 5e-5. In other words, we are likely to discover 13 out of 100 variants having the parameters considered at the significance level 0.05 but only 0.06 at the level 5e-5.

2. Effect size of 0.4, MAF of 10%, and 14k SNPs

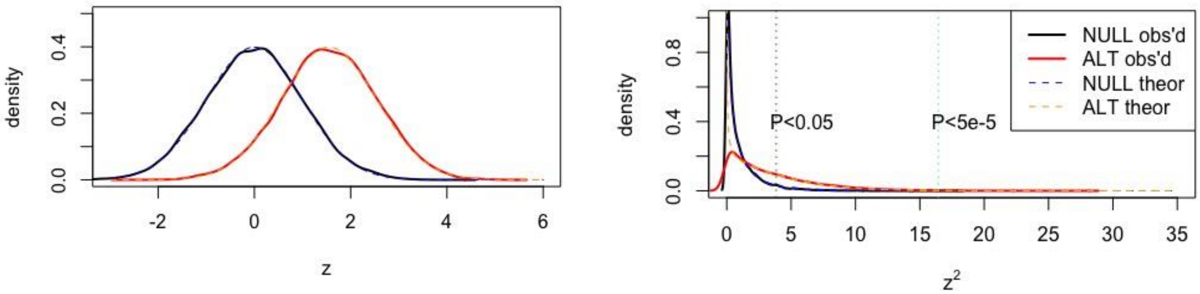

Interpretation of the distributions: theoretical and observed distributions match, but the alternative distribution move towards the right compared to previous simulation. We would discover more variants.

Interpretation of the proportions at the right of a given threshold*:* the significance threshold The significance threshold would be 0.339660899 0.006042161. We would have about 34% probability to detect a variant at significance threshold of 0.05, but still only 0.06 at 5e-5.

3. Effect size of 0.6 MAF of 10%, and 14k SNPs

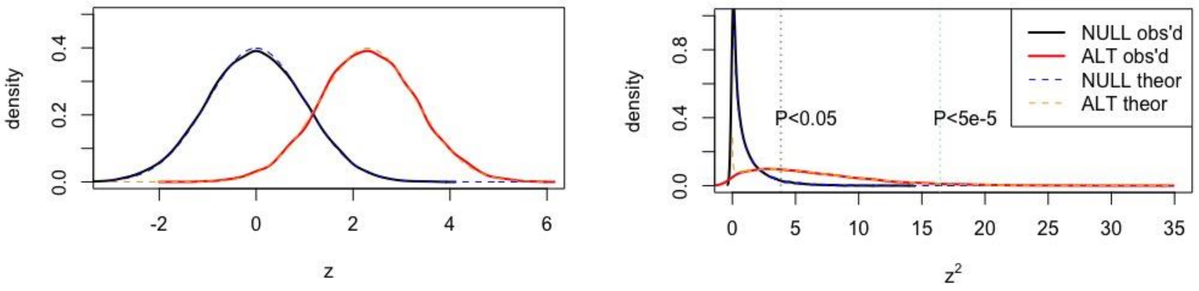

Interpretation of the distributions: theoretical and observed distributions match, now the alternative distribution is mostly the. We would discover most of the variants.

Interpretation of the proportions at the right of a given threshold*:* the significance threshold The significance threshold would be 0.63615885 0.04027714. We would have about 64% probability to detect a variant at significance threshold of 0.05, and about 40% at 5e-5.

Summary

	p value thresholds					
	Exponential		Decimal		-Log10 (Manhattan plot)	
Effect size	Lower	Higher	Lower	Higher	Lower	Higher
0.2	1.33E-01	6.37E-04	0.1326403	0.0006372	0.88	3.20
0.4	3.40E-01	6.04E-03	0.3396609	0.0060422	0.47	2.22
0.6	6.36E-01	4.03E-02	0.6361589	0.0402771	0.20	1.39

Conclusion

Although we do not know the variants true effect, we tested values varying from 0.2 to 0.6. It is worth to remember that a single *kdr* mutation results in a complete resistant phenotype with a huge effect size. We were able to find two variants above the significance threshold of 5e-5 and after correction of multiple testing. These loci and others that we did not have the power to detect may be acting synergistically with *kdr* mutations that do not confer completely resistance to pyrethroids, helping to detoxify the neurons from the insecticide. A higher sample size and more variants is necessary to completely rule out the contribution of other genes towards resistance.

Reference

Benjamini, Y., and Y. Hochberg, 1995 Controlling the False Discovery Rate - a Practical and Powerful Approach to Multiple Testing. Journal of the Royal Statistical Society Series B-Statistical Methodology 57**:** 289-300.

Pirinen, M., 2020 GWAS 3: Statistical Power, pp. https://www.mv.helsinki.fi/home/mjxpirin/GWAS_course/material/GWAS3.html

Sham, P. C., and S. M. Purcell, 2014 STUDY DESIGNS Statistical power and significance testing in large-scale genetic studies. Nature Reviews Genetics 15**:** 335-346.
