# Supplementary material for: Genome-wide Association Study Reveals New Loci Associated With Pyrethroid Resistance in Aedes aegypti
Source: Front Genet. 2022 Apr 11;13:867231. doi: 10.3389/fgene.2022.867231 (PMC9035894; doi:10.3389/fgene.2022.867231)
Supplement: Supplementary file 9 [file DataSheet2.docx]

**Supplemental Tables**

**Supplemental Table 1.** Primer and probe sequences for the SNPs V410L, V1016I, and F1534C *kdr* in *Aedes aegypti*.

| ***vgsc* site** | **Assay ID^1^** | **Variation** | **Primers** | **Probes** |
| --- | --- | --- | --- | --- |
| 1016 | AHS1DL6 | GTA/ATA | For: CGTGCTAACCGACAAATTGTTTCC | Val: VIC-CCCGCACAGGTACTTA-FAM |
|  |  | (Val/Ile) | Rev: GACAAAAGCAAGGCTAAGAAAAGGT | Ile: FAM-CCGCACAGATACTTA-NFQ |
| 1534 | AHWSL61 | TTC/TGC | For: TCGCGAGACCAACATCTACATG | Phe: VIC-AACGACCCGAAGATGA-NFQ |
|  |  | (Phe/Cys) | Rev: GATGATGACACCGATGAACAGATTC | Cys: FAM-ACGACCCGACGATGA-NFQ |

^1^Identification of the customized TaqMan SNP Genotyping Assay (Thermo Fischer)

**Supplemental Table 2.** List of genotypes based on SNP reactions for 1016 and 1534 Na_V_ sites of the *Aedes aegypti* populations.

| **1016** | **1534** | **Genotype** |
| --- | --- | --- |
| Val/Val | Phe/Phe  Phe/Cys  Cys/Cys | SS  SR1  R1R1 |
| Val/Ile | Phe/Phe  Phe/Cys  Cys/Cys | SR2  R1R2 |
| Ile/Ile | Phe/Phe  Phe/Cys  Cys/Cys | R2R2 |

**Supplemental Table 3**. Survivability and mortality of mosquitoes (number) after exposure to papers impregnated with deltamethrin. We characterized mosquitoes into three phenotypes: resistant (alive of 1-hour exposure), knockdown resistance (mosquitoes were knocked down after exposure but recovered 24 hours later), and susceptible (mosquitoes died after 1 hour of exposure).

| Population: Oiapoque - 1,200 mg/L | | | | |
| --- | --- | --- | --- | --- |
| Tube | Resistant (alive after 1-hour exposure) | Knockdown resistance (active at 24 hours) | Susceptible (dead at 24 hours) | Total |
| 1 | 40 | 0 | 14 | 54 |
| 2 | 41 | 0 | 12 | 53 |
| 3 | 45 | 2 | 11 | 58 |
| Total | 126 | 2 | 37 | 165 |
| Frequency | 0.76 | 0.01 | 0.22 |  |
| Population: Macapa - 600 mg/L | | | | |
| 1 | 8 | 0 | 38 | 46 |
| 2 | 19 | 5 | 51 | 75 |
| 3 | 4 | 11 | 43 | 58 |
| Total | 31 | 16 | 132 | 179 |
| Frequency | 0.17 | 0.09 | 0.74 |  |

**Supplemental Table 4**. Top 10 SNPs with the lowest *p* values before and after correction for multiple tests using Plink.

| Chromosome | SNP | Unadjusted *p-value* | BONF^1^ | FDR_BH^2^ |
| --- | --- | --- | --- | --- |
| 2 | AX-93253438 | 7.51E-07 | 0.01049 | 0.01049 |
| 3 | AX-93227955 | 8.14E-06 | 0.1137 | 0.05684 |
| 3 | AX-93248379 | 4.14E-05 | 0.578 | 0.1927 |
| 3 | AX-93226989 | 0.0001417 | 1 | 0.4948 |
| 2 | AX-93254599 | 0.0002112 | 1 | 0.5424 |
| 2 | AX-93237656 | 0.0002927 | 1 | 0.5424 |
| 3 | AX-93230051 | 0.0003181 | 1 | 0.5424 |
| 1 | AX-93260713 | 0.0003453 | 1 | 0.5424 |
| 3 | AX-93249913 | 0.0004334 | 1 | 0.5424 |
| 2 | AX-93252288 | 0.0004487 | 1 | 0.5424 |

^1^Bonferroni correction.

^2^FDR-Benjamini-Hochberg correction.

**Supplemental Table 5**. Top 10 SNPs with the lowest *p* values using CGTA.

| Chromosome | SNP | bp | A1 | A2 | Freq | Unadjusted *p-value* |
| --- | --- | --- | --- | --- | --- | --- |
| 2 | AX-93253438 | 92765528 | C | T | 0.371951 | 2.58E-06 |
| 3 | AX-93227955 | 84807303 | A | C | 0.335294 | 1.46E-05 |
| 2 | AX-93239189 | 421076044 | T | G | 0.150602 | 0.000443847 |
| 2 | AX-93230494 | 33412034 | T | C | 0.292135 | 0.000626716 |
| 3 | AX-93249913 | 149260415 | C | A | 0.247126 | 0.000641291 |
| 3 | AX-93252975 | 239519102 | T | C | 0.377778 | 0.000739477 |
| 3 | AX-93228410 | 253226724 | G | T | 0.151685 | 0.000825366 |
| 2 | AX-93239523 | 422623322 | T | C | 0.095238 | 0.000827654 |
| 3 | AX-93226989 | 179834611 | T | C | 0.344444 | 0.00085569 |
| 2 | AX-93240422 | 291575194 | G | A | 0.409639 | 0.00100276 |

**Supplemental Table 6**. Top 10 SNPs with the lowest *p* values from the epistasis analyses using Plink. The locus AX-93252255 is associated with pyrethroid resistance. The other loci were not significant in our association analyses.

| CHR1 | SNP1 | CHR2 | SNP2 | BETA_INT | STAT | P |
| --- | --- | --- | --- | --- | --- | --- |
| 3 | AX-93252255 | 2 | AX-93237274 | -1.4095 | 43.8707 | 3.53E-11 |
| 3 | AX-93231097 | 3 | AX-93256333 | -0.868218 | 42.5932 | 6.77E-11 |
| 2 | AX-93220153 | 2 | AX-93231531 | 1.1025 | 41.3107 | 1.31E-10 |
| 3 | AX-93229850 | 1 | AX-93241245 | 1.14773 | 40.6732 | 1.81E-10 |
| 2 | AX-93220153 | 2 | AX-93258299 | 1.18733 | 38.7463 | 4.85E-10 |
| 3 | AX-93251425 | 2 | AX-93263498 | 1.06473 | 37.5905 | 8.76E-10 |
| 3 | AX-93233129 | 2 | AX-93240785 | 1.33519 | 37.586 | 8.78E-10 |
| 2 | AX-93220152 | 2 | AX-93228918 | 1.26969 | 37.5053 | 9.15E-10 |
| 2 | AX-93220153 | 2 | AX-93233114 | 1.16601 | 37.0701 | 1.14E-09 |
| 2 | AX-93220153 | 2 | AX-93233119 | 1.16601 | 37.0701 | 1.14E-09 |

**Supplemental Table 7.** Compound genotypes frequencies of loci AX-9325343 + AX-93227955 according to their phenotype after insecticide exposure. We show mosquitoes where both loci were genotyped. A plot with the data is shown in Supplemental Figure 5.

| Genotype | Phenotype (number of mosquitoes) | | |
| --- | --- | --- | --- |
|  | Susceptible | Knockdown | Resistant |
| TT/CC | 11 | 4 | 9 |
| TT/AC | 10 | 0 | 5 |
| TT/AA | 2 | 0 | 0 |
| CT/CC | 5 | 1 | 0 |
| CT/AC | 1 | 0 | 0 |
| CT/AA | 1 | 0 | 0 |
| CC/CC | 0 | 0 | 0 |
| CC/AC | 3 | 9 | 11 |
| CC/AA | 0 | 1 | 0 |
| Total | 33 | 15 | 25 |

**Supplemental Table 8.** Compound genotypes frequencies of loci AX-9325343 + AX-93227955 according to their *kdr* genotype by qPCR after insecticide exposure. Only showing mosquitoes where both loci were genotyped. Now showing mosquitoes if a locus’ genotype was missing, either by SNP chip or qPCR. A plot with the data is shown in Supplemental Figure 6.

| Genotype | *kdr* genotype frequency (number of mosquitoes) | | | | | |
| --- | --- | --- | --- | --- | --- | --- |
|  | SS | SR1 | SR2 | R1R1 | R1R2 | R2R2 |
| TT/CC | 0 | 8 | 1 | 9 | 6 | 1 |
| TT/AC | 1 | 3 | 1 | 8 | 2 | 0 |
| TT/AA | 0 | 0 | 0 | 2 | 0 | 0 |
| CT/CC | 0 | 0 | 0 | 3 | 3 | 0 |
| CT/AC | 0 | 0 | 0 | 1 | 0 | 0 |
| CT/AA | 0 | 0 | 0 | 1 | 0 | 0 |
| CC/CC | 0 | 0 | 0 | 0 | 0 | 0 |
| CC/AC | 0 | 1 | 3 | 3 | 11 | 5 |
| CC/AA | 0 | 0 | 0 | 0 | 1 | 0 |
| Total | 1 | 12 | 5 | 27 | 23 | 6 |

**Supplemental Table 9.** Genotypes of the mosquitoes used in our study. The *kdr* genotypes were generated using qPCR.

| ID | Population | *kdr* | AX-93253438 | AX-93227955 | Resistance | Phenotypes |
| --- | --- | --- | --- | --- | --- | --- |
| 182 | Oiapoque | R1R2 | TT | CC | Susceptible | 1 |
| 183 | Oiapoque | R1R2 | CT | CC | Susceptible | 1 |
| 184 | Oiapoque | R1R1 | TT | CC | Susceptible | 1 |
| 185 | Oiapoque | R1R1 | TT | CC | Susceptible | 1 |
| 186 | Oiapoque | R1R2 | TT | AC | Susceptible | 1 |
| 187 | Oiapoque | R1R2 | Missing | CC | Susceptible | 1 |
| 188 | Oiapoque | SR1 | TT | CC | Susceptible | 1 |
| 189 | Oiapoque | R1R2 | TT | CC | Susceptible | 1 |
| 190 | Oiapoque | SR2 | CC | AC | Susceptible | 1 |
| 191 | Oiapoque | R1R1 | TT | CC | Susceptible | 1 |
| 194 | Macapa | R1R2 | TT | AC | Susceptible | 1 |
| 195 | Macapa | R1R2 | CT | CC | Susceptible | 1 |
| 196 | Macapa | R1R2 | TT | CC | Susceptible | 1 |
| 197 | Macapa | R1R2 | TT | CC | Susceptible | 1 |
| 198 | Macapa | SR2 | TT | AC | Susceptible | 1 |
| 199 | Macapa | R1R2 | CT | CC | Susceptible | 1 |
| 200 | Macapa | R1R1 | TT | CC | Susceptible | 1 |
| 201 | Macapa | SR2 | Missing | CC | Susceptible | 1 |
| 202 | Macapa | R1R1 | TT | CC | Susceptible | 1 |
| 203 | Macapa | R1R2 | TT | CC | Susceptible | 1 |
| 204 | Macapa | R1R1 | TT | CC | Susceptible | 1 |
| 205 | Macapa | R1R2 | TT | CC | Susceptible | 1 |
| 232 | Macapa | R1R1 | CT | Missing | Susceptible | 1 |
| 233 | Macapa | SR1 | TT | CC | Susceptible | 1 |
| 234 | Macapa | R1R1 | CT | CC | Susceptible | 1 |
| 235 | Macapa | SR1 | TT | CC | Susceptible | 1 |
| 236 | Macapa | SS | TT | AC | Susceptible | 1 |
| 237 | Macapa | SR1 | TT | AC | Susceptible | 1 |
| 238 | Macapa | R1R1 | TT | AA | Susceptible | 1 |
| 239 | Oiapoque | R1R1 | TT | AC | Susceptible | 1 |
| 240 | Oiapoque | SR1 | CT | AC | Susceptible | 1 |
| 241 | Oiapoque | SR1 | TT | CC | Susceptible | 1 |
| 242 | Oiapoque | SR1 | TT | AC | Susceptible | 1 |
| 243 | Oiapoque | SR1 | TT | AC | Susceptible | 1 |
| 244 | Oiapoque | SR1 | TT | CC | Susceptible | 1 |
| 245 | Oiapoque | R1R1 | CT | Missing | Susceptible | 1 |
| 246 | Oiapoque | R1R1 | TT | CC | Susceptible | 1 |
| 247 | Oiapoque | SR1 | TT | CC | Susceptible | 1 |
| 248 | Oiapoque | SR1 | TT | CC | Susceptible | 1 |
| 249 | Oiapoque | R1R1 | CT | AC | Susceptible | 1 |
| 250 | Oiapoque | SR1 | TT | CC | Susceptible | 1 |
| 251 | Macapa | R1R1 | TT | CC | Susceptible | 1 |
| 252 | Macapa | R1R1 | TT | AC | Susceptible | 1 |
| 253 | Macapa | R1R1 | TT | CC | Susceptible | 1 |
| 254 | Macapa | SR1 | Missing | AC | Susceptible | 1 |
| 255 | Macapa | R1R1 | TT | AC | Susceptible | 1 |
| 157 | Oiapoque | R1R2 | CC | AC | Knockdown | 2 |
| 158 | Oiapoque | SR2 | TT | CC | Knockdown | 2 |
| 219 | Macapa | R1R1 | Missing | AA | Knockdown | 2 |
| 222 | Macapa | R1R1 | TT | AC | Knockdown | 2 |
| 223 | Macapa | R1R1 | TT | AC | Knockdown | 2 |
| 224 | Macapa | R1R1 | Missing | AC | Knockdown | 2 |
| 225 | Macapa | R1R1 | CT | Missing | Knockdown | 2 |
| 227 | Macapa | R1R1 | CC | AC | Knockdown | 2 |
| 228 | Macapa | R1R1 | CT | AC | Knockdown | 2 |
| 229 | Macapa | R1R1 | TT | AA | Knockdown | 2 |
| 230 | Macapa | SR1 | CC | AC | Knockdown | 2 |
| 231 | Macapa | R1R1 | CT | AC | Knockdown | 2 |
| 277 | Macapa | R1R1 | CT | Missing | Knockdown | 2 |
| 278 | Macapa | SR1 | Missing | AC | Knockdown | 2 |
| 279 | Macapa | R1R1 | CC | AC | Knockdown | 2 |
| 159 | Oiapoque | R2R2 | CC | AC | Resistant | 3 |
| 161 | Oiapoque | SR2 | CC | AC | Resistant | 3 |
| 162 | Oiapoque | R2R2 | CT | AC | Resistant | 3 |
| 163 | Oiapoque | R1R2 | CC | AA | Resistant | 3 |
| 164 | Oiapoque | R2R2 | CC | AC | Resistant | 3 |
| 166 | Oiapoque | R1R2 | CC | AC | Resistant | 3 |
| 170 | Oiapoque | R1R2 | CC | AC | Resistant | 3 |
| 171 | Oiapoque | R1R2 | CC | AC | Resistant | 3 |
| 172 | Oiapoque | R1R2 | CC | AC | Resistant | 3 |
| 173 | Oiapoque | R2R2 | CC | AC | Resistant | 3 |
| 207 | Macapa | R1R1 | CT | AC | Resistant | 3 |
| 208 | Macapa | R1R1 | CT | AC | Resistant | 3 |
| 209 | Macapa | R1R1 | CT | CC | Resistant | 3 |
| 211 | Macapa | R1R1 | CT | AA | Resistant | 3 |
| 212 | Macapa | R1R1 | CT | AC | Resistant | 3 |
| 214 | Macapa | R1R1 | CT | AC | Resistant | 3 |
| 215 | Macapa | R1R1 | TT | AC | Resistant | 3 |
| 216 | Macapa | SR1 | Missing | CC | Resistant | 3 |
| 217 | Macapa | R1R1 | CT | CC | Resistant | 3 |
| 218 | Macapa | R1R1 | TT | AC | Resistant | 3 |
| 257 | Oiapoque | Missing | CC | AC | Resistant | 3 |
| 258 | Oiapoque | SR2 | CC | AC | Resistant | 3 |
| 260 | Oiapoque | R2R2 | CC | AC | Resistant | 3 |
| 261 | Oiapoque | R1R2 | CC | AC | Resistant | 3 |
| 262 | Oiapoque | R1R2 | CC | AC | Resistant | 3 |
| 263 | Oiapoque | R2R2 | CC | AC | Resistant | 3 |
| 264 | Oiapoque | R2R2 | CC | Missing | Resistant | 3 |
| 265 | Oiapoque | R1R2 | CC | AC | Resistant | 3 |
| 266 | Oiapoque | R1R2 | CC | AC | Resistant | 3 |
| 267 | Oiapoque | R1R2 | CC | AC | Resistant | 3 |
| 270 | Oiapoque | SR2 | CT | AC | Resistant | 3 |
| 271 | Oiapoque | R1R2 | CC | AC | Resistant | 3 |
| 274 | Macapa | R1R1 | TT | AC | Resistant | 3 |
| 275 | Macapa | SR1 | Missing | AC | Resistant | 3 |

**Supplemental Table 10**. LDna summary statistics for each single outlier cluster (SOC).

| **Name** | **Type** | **Merge.at** | **nLoci** | **nE** | **lambda** | **Median.LD** | **MAD.LD** |  |
| --- | --- | --- | --- | --- | --- | --- | --- | --- |
| **OIAs** | | | | | | | | |
| 146_0.54 | SOC | 0.53 | 27 | 46 | 11.25 | 0.239 | 0.162 |  |
| 150_0.54 | SOC | 0.51 | 18 | 40 | 0.469 | 0.261 | 0.150 |  |
| 187_0.48 | SOC | 0.47 | 21 | 41 | 4.375 | 0.109 | 0.081 |  |
| **MACs** | | | | | | | | |
| 109_0.62 | SOC | 0.59 | 14 | 42 | 4.375 | 0.536 | 0.229 |  |
| 126_0.58 | SOC | 0.57 | 19 | 41 | 2.969 | 0.395 | 0.147 |  |
| 189_0.47 | SOC | 0.46 | 20 | 59 | 10.678 | 0.245 | 0.201 |  |
| 227_0.4 | SOC | 0.39 | 26 | 50 | 0.000 | 0.103 | 0.090 |  |
| **OIAr** | | | | | | | | |
| 127_0.62 | SOC | 0.6 | 12 | 40 | 17.500 | 0.622 | 0.268 |  |
| 132_0.61 | SOC | 0.6 | 36 | 59 | 7.500 | 0.149 | 0.117 |  |
| **MACr** | | | | | | | | |
| 146_0.48 | SOC | 0.46 | 33 | 77 | 2.579 | 0.137 | 0.128 |  |
| 187_0.42 | SOC | 0.41 | 27 | 50 | 2.8125 | 0.078 | 0.073 |  |
| 203_0.4 | SOC | 0.39 | 38 | 59 | 1.980 | 0.074 | 0.061 |  |

**Supplemental Table 11.** Genomic coordinates and size of each single outlier cluster (SOC) bigger than 1Mb on chromosome 3.

| **Name** | **Start** | **End** | **Size (Mb)** |
| --- | --- | --- | --- |
| **OIAs** | | | |
| 146_0.48 | 22,936,653 | 117,618,630 | 94 |
|  | 202,454,345 | 206,631,260 | 4 |
| 150_0.54 | 217,539,374 | 224,063,946 | 6 |
|  | 251,067,253 | 361,396,176 | 110 |
| 187_0.48 | 208,024,078 | 216,898,404 | 8 |
| **MACs*** | | | |
| 126_0.58 | 202,454,345 | 218,246,334 | 15 |
| 189_0.47 | 113,388,262 | 115,917,923 | 2 |
| 227_0.4 | 28,541,353 | 107,192,381 | 78 |
|  | 119,566,705 | 122,190,621 | 2 |
|  | 234,494,421 | 236,875,627 | 2 |
|  | 246,835,391 | 355,123,121 | 108 |
| **OIAr**** | | | |
| 132_0.61 | 100,248,557 | 114,275,875 | 14 |
|  | 203,078,039 | 204,094,265 | 1 |
|  | 207,110,297 | 211,138,027 | 4 |
|  | 211,833,659 | 226,695,452 | 14 |
| **MACr** | | | |
| 146_0.48 | 110,941,767 | 112,606,575 | 1 |
|  | 113,388,262 | 123,316,182 | 9 |
| 187_0.42 | 205,940,682 | 207,121,057 | 1 |
| 203_0.4 | 217,539,374 | 223,010,464 | 5 |
|  | 223,314,434 | 254,285,891 | 30 |
|  | 348,735,969 | 359,041,763 | 10 |

* Cluster 109_0.62 is smaller than 1Mb and therefore not shown on this table.

** Cluster 127_0.62 is smaller than 1Mb and therefore not shown.
